# Mixed infections by different *Trypanosoma cruzi* discrete typing units among Chagas disease patients in an endemic community in Panama

**DOI:** 10.1371/journal.pone.0241921

**Published:** 2020-11-12

**Authors:** Alexa Prescilla Ledezma, Roberto Blandon, Alejandro G. Schijman, Alejandro Benatar, Azael Saldaña, Antonio Osuna

**Affiliations:** 1 Institute of Biotechnology, Department of Parasitology, University of Granada, Granada, Spain; 2 Center for Research and Diagnosis of Parasitic Diseases (CIDEP), Faculty of Medicine, University of Panama, Panama, Panama; 3 Cardiology Service, Santo Tomas Hospital, Panama, Panama; 4 Laboratory of Molecular Biology of Chagas Disease, Institute of Research in Genetic Engineering and Molecular Biology “Dr Héctor Torres” (INGEBI-CONICET), Buenos Aires, Argentina; 5 Gorgas Memorial Institute of Health Studies (ICGES), Panama, Panama; Instituto Rene Rachou, BRAZIL

## Abstract

**Background:**

*Trypanosoma cruzi*, the hemoparasite that causes Chagas disease, is divided into six Discrete Typing Units or DTUs: TcI-TcVI plus Tcbat. This genetic diversity is based on ecobiological and clinical characteristics associated with particular populations of the parasite. The main objective of this study was the identification of DTUs in patients with chronic chagasic infections from a mountainous rural community in the eastern region of Panama.

**Methods:**

A total of 106 patients were tested for Chagas disease with three serological tests (ELISA, rapid test, and Western blot). Molecular diagnosis and DTU typing were carried out by conventional PCRs and qPCR targeting different genomic markers, respectively. As a control sample for the typing, 28 patients suspected to be chagasic from the metropolitan area of Panama City were included.

**Results:**

Results showed a positivity in the evaluated patients of 42.3% (33/78); high compared to other endemic regions in the country. In the control group, 20/28 (71.43%) patients presented positive serology. The typing of samples from rural patients showed that 78.78% (26/33) corresponded to TcI, while 9.09% (3/33) were mixed infections (TcI plus TcII/V/VI). Seventy-five percent (15/20) of the patients in the control group presented TcI, and in five samples it was not possible to typify the *T*. *cruzi* genotype involved.

**Conclusions:**

These results confirm that TcI is the main DTU of *T*. *cruzi* present in chronic chagasic patients from Panama. However, the circulation of other genotypes (TcII/V/VI) in this country is described for the first time. The eco-epidemiological characteristics that condition the circulation of TcII/V/VI, as well as the immune and clinical impact of mixed infections in this remote mountainous region should be investigated, which will help local action programs in the surveillance, prevention, and management of Chagas disease.

## Introduction

Chagas disease or American trypanosomiasis is a zoonosis spread from the southern USA to approximately the Río Negro in Argentina, whose causative agent is the hemoparasite *Trypanosoma cruzi* [1]. This infection is transmitted to humans through different routes: i) by direct contamination of the mucosa with the feces of the vector triatomine insects; ii) by ingesting food contaminated with the feces of the vectors; iii) by congenital vertical transmission, and iv) by blood transfusions or transplants of infected organs [1–4]. Currently, the estimated number of patients infected with *T*. *cruzi* is 6–8 million and 120 million people are at risk of infection. Chagas disease is the most important parasitic disease in Latin America and one of the most common neglected diseases worldwide [5, 6]. The disease is characterized by an acute phase that occurs in most cases asymptomatically or with nonspecific symptoms such as fever, malaise and hepatomegaly [7, 8]. Mortality during this acute phase is low (<5%) and mostly linked to myocarditis or meningoencephalitis [9, 10]. Approximately two months after the onset of infection, the chronic phase starts in 70% of the patients and there may be no symptoms even after many years of evolution [11, 12]. The remaining 30% of chronic patients develop heart disease of different degrees, representing the majority of deaths due to Chagas disease [4, 13]. In addition, 10% of these patients may also develop digestive alterations such as megacolon and megaesophagus as well as neurological alterations or a combination of both [4, 14, 15].

One of the possible causes of the diversity of the clinical manifestations of Chagas disease has been attributed to the high genetic variability and the multiclonal character of the natural populations of *T*. *cruzi*. [16]. This diversity has been demonstrated by different research groups based on the marked variety among the different clones of the parasite [17]. Among the differences studied, histotropism, intracellular invasion and multiplication capacity, vector infectivity, drug sensitivity, morphology of trypomastigote forms, antigenic profile and potential association to pathologies are mentioned. Different genotypic markers have allowed the current identification of six discrete typing units, or DTUs, among the different *T*. *cruzi* isolates plus a different variant identified as TcBat [18]. The enormous genetic variability found in this species favors the appearance of these "strains", which complicates the classification according to their pathology or other phenotypic peculiarities [19].

The geographical distribution of DTUs is particular, as well as their link with reservoirs, transmission cycles, and diagnostic methods [16, 20, 21].

TcI is currently considered one of the parasite's parental lineages [22]. Geographically, TcI predominates in the Andes region, in Central and North America, Mexico [23–25], presenting a sylvatic transmission cycle, mainly between marsupials and sylvatic triatomines [26–28]. This DTU is also associated with human infections reported from the United States [29], Mexico, Central America [30, 31], and northern South America, having been detected in patients with cardiomyopathies from Venezuela [32] and Colombia [33–37].

DTUs (II, III, IV and V) have been found in vector insects, both in Colombia and Venezuela as well as in the rest of South America [38, 39]. DTU II has been described as the cause of parasitation in humans in both acute and chronic cases, as well as in vectors from Colombia, and this DTU is considered to correspond to a primarily domiciliary cycle [40, 41].

Chagasic infection has been known in Panama since 1930 [42, 43]. In 2005, the Pan-American Health Organization (PAHO) estimated, based on official data, that the seroprevalence in this country was 0.01% [44]. Subsequent studies located the rural regions near the Panama Canal as the areas with the highest number of cases [43, 45, 46]. Recent research confirmed that the infection is also common in other areas located west and east of the capital city [47–49]. In contrast, there are still remote rural communities with the appropriate eco-epidemiological conditions for the transmission of Chagas disease where correct epidemiological surveillance has not been implemented [49–51]. From 2007 to 2017, an average of 56 annual cases of Chagas disease were reported in Panama, for a total population of about 4,159,000 inhabitants [52]. Although these figures are below those reported in other countries of Central and South America [49], chagasic infection in Panama is still an important public health concern [53]. In Panama, the main Chagas disease vector is *Rhodnius pallescens*, a triatomine present in the *Attalea butyracea* palm, which is often found in peridomestic areas of endemic communities and that invades nearby dwellings, but does not settle inside them [37, 54–56]. On the other hand, the clinical characteristics related to Chagas disease in Panama involve heart diseases similar to those described in other regions of the continent [25, 57, 58]. However, until now no clinical cases of Chagas disease with digestive pathologies have been described in Panama [42, 59].

The genetic variants of circulating *T*. *cruzi* in vectors and acute patients have been partially investigated in Panama [25, 49]. Yet, in these preliminary studies, only *T*. *cruzi* TcI was detected [57, 60] as well as the typification of a *T*. *cruzi* isolate from a rural area of the Arraiján district [17].

Currently, there are no investigations being carried out aimed at the identification of *T*. *cruzi* DTUs from blood samples from Panamanian patients. This type of study reflects the genetic diversity of parasites in natural infections without the need for their isolation or expansion, a particularly difficult situation in chronic patients with very low parasitemias [57, 61].

Moreover, genotyping of cultured parasite isolates may lead to misidentification of minor genotypes in mixed infections, since populations that grow at a higher rate are more likely to be detectable by the available typing approaches.

Although information on the distribution of DTUs in different geographical areas is increasing, confirmed association with the clinical manifestations and epidemiology of Chagas disease is limited. As there is a new focus of Chagas disease in Panama, it is relevant to propose ecobiological studies of *T*. *cruzi* infection in this region of the country aiming to improve surveillance and control measures.

The main objective of the study was the direct identification of *T*. *cruzi* DTUs in blood samples from chronic chagasic patients resident in this new highly endemic rural community, “Las Margaritas”, Chepo district, Panama province, in comparison to the DTUs detected in Chagas disease patients in the metropolitan area of Panama City, representative of *T*. *cruzi* infected people in the main endemic regions of the country.

## Materials and methods

### Ethical statement

All protocols involving human subjects were approved by the Bioethics Committee of Santo Tomás Hospital in Panama City and were assigned the identification code No. 2015–310 V1. A total of 106 enrolled patients signed written or oral informed consent forms. Individuals who could not read, stamped their fingerprint on the document, in the presence of two witnesses. Acceptance of informed consent was carried out before the study activities were initiated and the analysis was performed according to the approved guidelines. Individuals over 18 years of age signed the consent form and responded to the social and epidemiological questionnaire. Parents and/or guardians of children and youth under 18 years of age provided their written consent for the collection of blood and responded to the social and epidemiological questionnaire.

### Patients and sampling

**Fig 1:**
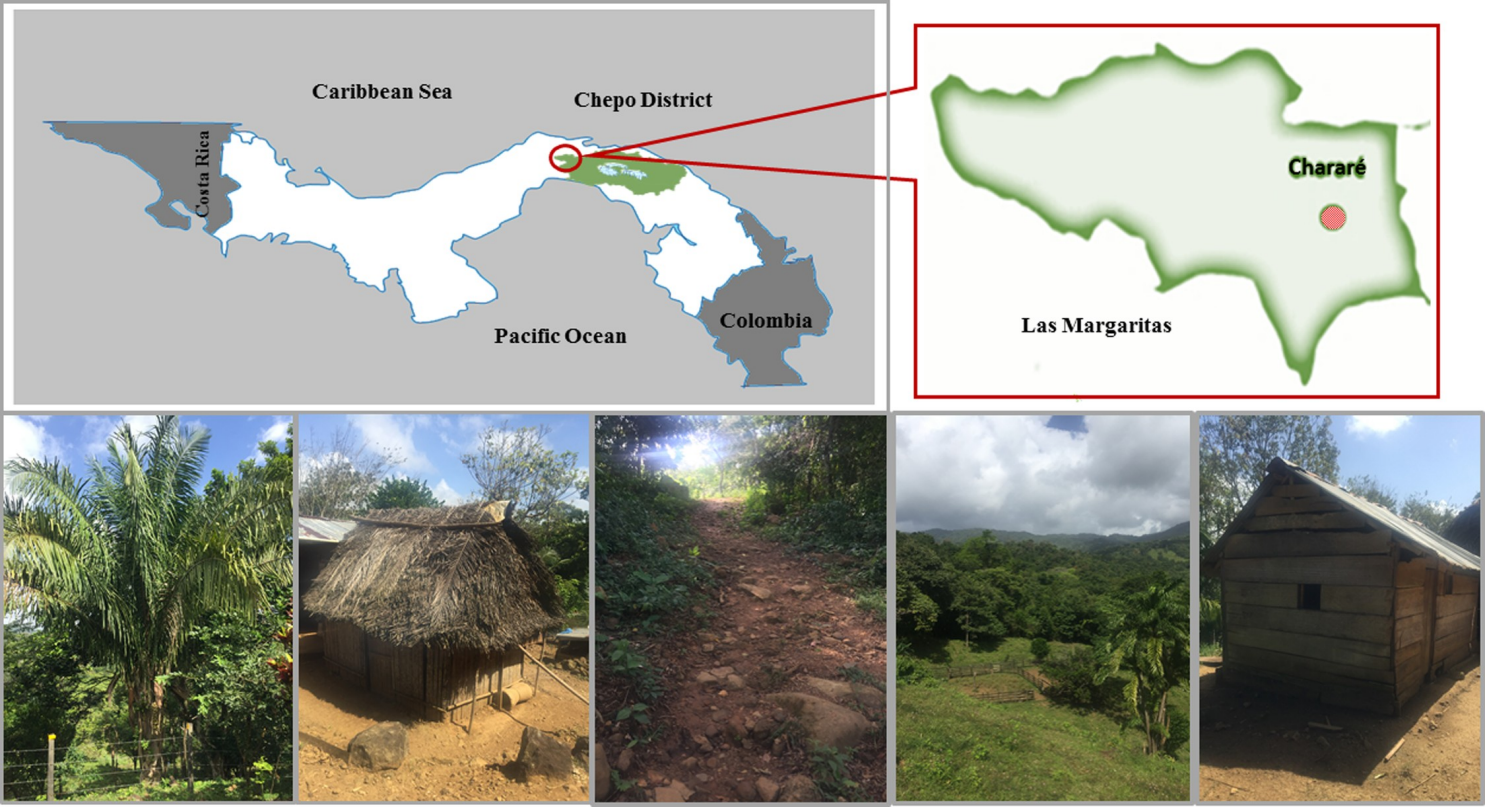
The locations of seropositive patients: Chararé community, Las Margaritas district, Chepo district, Panama province. The Map has been drawn by the authors, as well as the photos that are original.

The study was carried out between April 2016 and January 2017 in two different epidemiological settings: 1) a small rural community (Charare), located in a mountainous area, in the township of Las Margaritas, Chepo district, Panama province (coordinates 9.243640, -79.059162) (Fig 1).

The total population of Charare is approximately 250 inhabitants. Sampling was done for convenience; all inhabitants were invited to participate through messages on a local radio station. The total number of samples studied was 106; 73.58% (78/106) corresponding to residents of Charare and 2) a total of 28 suspected chagasic patients diagnosed in the metropolitan area of Panama City, of whom 20/28 (71.42%) presented positive serology. All these patients also signed the informed consent form. This group of patients represents the *T*. *cruzi* infected population from the main endemic regions of the country, as many of whom travelled to Panama City to be admitted to hospitals. Therefore, their parasitic populations are representative of the main circulating genetic variants of the parasite in the country. All hospitalized cases with positive serology had been diagnosed due to their clinical manifestations: repolarization disorders, atrioventricular block, bradycardia, and conduction disorder. The diagnoses were made in the tertiary care hospitals included in the study (Santo Tomás Hospital, Dr. Arnulfo Arias Madrid Hospital Complex, and Omar Torrijos Herrera Hospital for Pediatric Specialties), using ARCHITECT System® Chagas assay (Abbott, Germany). Reconfirmation of *T*. *cruzi* infection was carried out in all rural and urban cases, using at least two immunological tests as described below. All urban patients had undergone specialized medical evaluation in the respective hospitals. In the case of patients from the rural community, once the serological diagnosis was made, they were clinically evaluated at the Regional Hospital of Chepo district, where they were evaluated and treated according to the clinical characteristics found.

The total number of patients with positive serology evaluated in the study was 53, and 20 of whom were treated at third-level hospitals in Panama City distributed as follows: 13 at the Cardiology service of Santo Tomás Hospital, four diagnosed at the Blood Bank of the Social Security Fund and two at the Infectology Outpatient Clinic of the Dr. Arnulfo Arias Madrid Hospital Complex of the Social Security Fund.

Thirty-three of the seropositive patients corresponded to the rural areas, all of whom permanent residents in the community of Chararé.

All individuals who agreed to participate in the study had two venous blood samples taken. The first sample of approximately 5 ml was collected in a Vacutainer™ EDTA vacuum blood collection tube for molecular assays. The blood sample was mixed 1:1 with a 6M guanidine hydrochloride solution (Sigma, St. Louis, USA.) and 200 mM EDTA, pH 8.0 (GEB) [62]. These samples were kept at laboratory temperature (22° C) for at least 4 days. For the second sample, BD Vacutainer ™ Venous Blood Collection Tubes were used (Vacutainer Plus ™ Glass Serum Tube), and 5 ml of blood were taken. After the clot formed, each tube was centrifuged at 1,500 x *g* for 10 min and approximately 2.0 ml of serum were collected to carry out the different serological tests.

### Serological tests

The presence of anti-*T*. *cruzi* antibodies in the different samples was confirmed by commercial tests of recombinant ELISA Chagatest, Wiener Lab, Argentina. Briefly, 10 μL of each serum sample were used and diluted with 200 μL of the sample diluent, then incubated for 30 min at 37°C, and washed 5 times with diluted 1:5 washing buffer. Next, 50 μL of the conjugate (peroxidase-conjugated anti-human (goat) immunoglobulins) were added and incubated for 30 minutes at 37°C. Subsequently, 5 washes were carried out with 300 μL thereof and the residue was completely aspirated. After the washings were completed, 50 μL of each of the developers were added: A (hydrogen peroxide 60 mmol / l in citrate buffer 50 mmol / L pH 3.2) and B (tetramethylbenzidine (TMB) 0.01 mol / l in 0.1 N hydrochloric acid) by incubating the wells for 30 minutes at room temperature. Finally, 50 μL of stop solution (2N sulfuric acid) were added and the reading was carried out at 490 nm in a Multiskan® Spectrum reader (Thermo Fisher Scientific, Finlandia). Both negative (non-reactive, inactivated serum dilution) and positive (inactivated serum dilution containing antibodies against *T*. *cruzi*) samples were performed in triplicate.

The SD Bioline Chagas AB Rapid test (Abbott, Germany) was used, following the manufacturer’s instructions. Briefly, 100 μL of the study serum were added to the sample well and 50 μL of the assay buffer (100 mM Tris-CI (5 ml) were added. It was then incubated at room temperature for 15 minutes. The test was read visually after 15 minutes. Any positive sample with the Chagas Ab SD Bioline Rapid Test was confirmed with alternate test methods. As a complementary assay, an immunoblot test of a Panamanian isolate of *T*. *cruzi* was used following the methodology described [63]. Briefly, each membrane was incubated with a 1:100 dilution (in milk solution) of the sera of the evaluated patients. Incubation was carried out under shaking, at room temperature, overnight, in 2.5ml tubes containing the nitrocellulose strip and 700μl of the respective serum dilution. The strip was then washed 3 times with PBS and incubated with human anti-immunoglobulin conjugate (DAKO IgG, IgM and IgA) diluted at 1:700 in PBS for one hour. Then, each strip was washed 3 times with PBS, before adding the substrate-chromogen (H_2_O_2_, PBS, NiCl, diaminobenzidine); the reaction was stopped with tap water, after approximately 10 minutes. The strips were photographed for the interpretation of the generated profiles.

The patients were considered positive when they showed positivity in at least two of the three immunological tests.

A questionnaire containing demographic information and indicators of knowledge, attitudes and risk factors for the transmission of Chagas disease as well as the mode of transmission, main clinical characteristics and biology of the vector (habits and ecotope, among others) was given to each patient with positive serology for chagasic infection **(S1 Questionnaire)**. An anonymous identification number was used to identify information in the questionnaires.

### DNA extraction

DNA extraction from blood samples was carried out using a commercially available kit (High Pure PCR Template preparation kit, Roche Applied Science), following the manufacturer's instructions. For the extraction of DNA, 500 μl of GEB were used and finally eluted in 100 μl ultrapure water. The extracted DNA was stored at -20°C until further analysis [64, 65].

### Polymerase chain reaction for the detection of *Trypanosoma cruzi*

*Trypanosoma cruzi* DNA detection was performed by PCR analysis in a CFX96 ™ Real-Time System thermal cycler CFX-96 Real Time System (Bio-Rad Laboratories). The reaction mixture was made with a 1X Go Taq® flexi buffer amplification buffer, 25 mM dNTPs, 2.5 mM MgCl2, 1.0 U of Go Taq® (Promega, USA), 10 pmol of specific primers 121 (AAATAATGTACGGGKGAGATGCATGA) and 122 (GGTTCGATTGGGGTTGGTGTAATATA) according to the methodology described [66]. The amplification conditions consisted of two cycles, each of 1 min at 98°C and 2 min at 64°C, followed by 33 cycles of 1 min at 94°C, and 1 min at 64°C, before a final extension at 72°C for 10 min. A DNA sample from *T*. *cruzi* tripomastigote forms in culture was used as a positive control, and a negative control was run without a DNA sample. PCR products were analyzed by agarose gel electrophoresis with SYBR Green and UV visualization in ChemiDoc MP Imaging System Bio Rad. A 330-bp product was considered indicative of the presence of the kinetoplastid DNA (kDNA) of *T*. *cruzi*.

### Genotyping assays of *T*. *cruzi* by PCR assays

For the identification of the *T*. *cruzi* DTUs detectable in the blood samples, a series of PCRs targeting different genomic markers was carried out, following the PCR-based algorithm reported by Burgos and co-authors [65], as described in S1 Table and S1 Fig. The DTU typing algorithm included the following *T*. *cruzi* genomic targets:

(1) the Spliced Leader intergenic region (SL-IR PCR) was used to distinguish *T*. *cruzi* I (150 bp), II, V, and VI (157 bp) from *T*. *cruzi* III and IV (200 bp); (2) first round and hemi- nested SL-IR-I was used to identify *T*. *cruzi* I DTUs (475 bp and 350 bp, respectively), and first round and hemi-nested SL-IR-II was used to identify *T*. *cruzi* II, V, and VI DTUs (425 bp and 300 bp, respectively); (3) hemi-nested PCR of 24S α- ribosomal DNA was used to distinguish *T*. *cruzi* V (125 or 125 + 140 bp) from *T*. *cruzi* II and VI (140 bp); and (4) hemi- nested real-time PCR targeting the A10 fragment was used to discriminate *T*. *cruzi* II (580 bp) from *T*. *cruzi* VI (525 bp). Samples that yielded positive SL-IR-II PCR results but negative 24S α-ribosomal DNA PCR results were reported as *T*. *cruzi* II, V, or VI (II/V/VI). Samples that amplified the 140-bp 24Sα-rDNA fragment but negative results of real-time PCR targeting A10 were reported as *T*. *cruzi* II or VI, as described [65].

## Results and discussion

Of the 78 rural patients evaluated, 42.3% (33/78) were seropositive for *T*. *cruzi* infection. This percentage suggests the existence of a high prevalence of chagasic infection in this rural area of Panama, when compared to the percentages found in recent studies in other endemic areas, where lower seroprevalences were demonstrated [48]. However, this high frequency of chagasic infection is consistent with sero-epidemiological studies carried out in other near endemic regions. As described by Anez et al. (2004), rural regions of Venezuela have a seropositivity of 56.8%, and in the Arauca region in Colombia, 21.6% of 0 to 14 year-old children tested positive for chagasic infection [32, 67, 68].

In 2010, the Department of Vector Control of the Ministry of Health of Panama reported the presence of the two main vectors of Chagas disease (*R*. *pallescens* and *Triatoma dimidiata*) in the community of Chararé. Around that time, the first cases of Chagas disease were diagnosed in the community. However, the genetic characteristics of circulating *T*. *cruzi* populations in the area have hitherto not been studied. This mountainous area is located in a remote region of difficult access, its inhabitants share low eco-epidemiological and socioeconomic characteristics that predispose the transmission of multiple pathogens, including *T*. *cruzi* (Table 1) [48].

**Table 1:** Sociodemographic characteristics of the interviewees by area of residence.

**Variable**	Total n = 53		Urban Patients n = 20		Rural Patients n = 33	
	**n**	**%**	**n**	**%**	**n**	**%**
**Sex**						
Female	25	47.2	7	40.0	18	54.5
Male	28	52.8	13	60.0	15	45.5
**Age**						
Less than 25 years	10	18.9	0	0.0	10	30.3
From 25 to 39 years	18	34.0	9	45.0	9	27.3
From 40 to 59 years	17	32.1	9	45.0	8	24.2
Over 60 years	8	15.0	2	10.0	6	18.2
Total	53	100.0	20	100.0	33	100.0
**Time of Residence**	Total n = 43		Urban Patients n = 20		Rural Patients n = 23	
Less Than 1 year	3	7.0	2	10.0	1	4.3
From 2 to 5 years	5	11.6	5	25.0	0	0.0
More than 5 years	35	81.4	13	65.0	22	95.7
**Education**						
No degree	5	11.6	0	0.0	5	21.7
Primary	21	48.8	7	35.0	14	60.9
High School	10	23.3	6	30.0	4	17.4
Tertiary	3	7.0	3	15.0	0	0.0
University superior	4	9.3	4	20.0	0	0.0
**Occupation**						
Employee	13	30.2	6	30.0	7	30.4
Self-employed	12	27.9	6	30.0	6	26.1
Domestic work	11	25.6	0	0.0	7	30.4
Unemployed	4	9.3	5	25.0	3	13.1
No answer	3	7.0	3	15.0	0	0.0
**Household residents**						
1	4	9.3	2	10.0	2	8.7
From 2 to 5	15	34.9	8	40.0	7	30.4
More than 5 people	24	55.8	10	50.0	14	60.9
**Patients in the home**						
1	38	88.4	20	100.0	18	78.3
2	3	7.0	0	0.0	3	13.1
3	1	2.3	0	0.0	1	4.3
4	1	2.3	0	0.0	1	4.3

All seropositive cases detected in the Chararé region can be considered chronic Chagas disease patients. Prior to sample collection, none of the patients reported fever or clinical signs that could suggest the acute phase of the disease. *T*. *cruzi* infection was characterized in these patients by means of a kDNA-based PCR test; 90% out of 20 urban patients were kDNA-PCR positive and 100% of patients from Chararé were kDNA-PCR positive. Genotyping of DTUs of these patients’ blood samples was carried out from the same DNA extracts without previous knowledge of the kDNA-PCR result. In fact, kDNA-PCR negative samples did not amplify genes used for DTU typing. Among kDNA-PCR positive samples, it was possible to identify DTUs in 15/18 and 29/33 samples from rural and urban localities (Table 2) and only after amplification of the *T*. *cruzi* SL-IR I and SL-IR II genes as molecular targets, whereas the other genotyping markers gave non-detectable results. Thus, distinction could only be made between TcI, and Tc II/V/VI DTU groups. These SL-IR I and II based PCR procedures showed that in Chararé, 78.78% of patients were infected with DTU I strains, while 9.09% corresponded to mixed DTU I plus DTU II/V/VI infections. In four of these samples (12.12%), the corresponding DTU group could not be determined (Table 2). The failure to genotype some of the tested samples was probably due to low parasitic loads, below the detection limits of the SL-IR I and II PCR methods (S1 Table) [65, 69]. The same limitation can be suspected for the other genotyping markers that gave undetectable findings.

**Table 2:** Serological and PCR-based findings in the two patient groups studied. Patients screened with the different methodologies in the two study groups. The presence of 5.6% (3/53) of patients with mixed DTU (DTU I and II/V/VI) is clearly presented.

Samples	Urban Patients	Rural Patients	Total
**Total tested samples**	28 (26.42%)	78 (73.58%)	106 (100%)
**Anti-*T*. *cruzi* seroreactivity**	20 (71.43%)	33 (42.30%)	53 (50%)
**kDNA-PCR positivity**	18/20 (90%)	33/33 (100%)	51/53 (96.22%)
**DTU PCR positivity**	15/20 (75%)	29/33 (87.88%)	44/53 (83.01%)
**Tc I**	15/20(75%)	26/33 (78.78%)	41/53 (77.35%)
**Mixed**	--	3/33 (9.09%)	3/53 (5.66%)*
**Tc I + Tc II/V/VI**			
**Untyped DTUs#**	5/20 (25%)	4/33 (12.12%)	9/51 (17.64%)

* The presence of 5.6% (3/53) of patients with mixed DTU (DTU I and II/V/VI) is clearly presented.

# The numbers include those cases with nondetectable kDNA-PCR findings plus those kDNA-PCR positive samples that gave nondetectable results in the PCRs performed for DTU genotyping.

The kDNA-PCR analysis of seropositive samples from urban chagasic patients, was positive in 18/20 (90%) of the samples. Fifteen of the samples, equivalent to 75%, were infected with Tc I populations and in five samples, it was not possible to typify the *T*. *cruzi* DTU involved. DTUs III/IV were not detectable in these populations. From the PCR positive data, it can be deduced that the analyzed blood samples from the chronic patients present circulating trypomastigote forms of the parasite as well as amastigote forms resulting from the death and rupture of infected cells, or there is DNA contamination from the kinetoplast of the parasite due to the destruction of the parasite forms and the consequent release of the kDNA. Among seropositive patients diagnosed in urban hospitals (ARCHITECT System Chagas assay), 71.42% (20/28) presented a clinical profile with heart disease evidenced by echocardiogram and chest radiography, and the data of rural endemic community patients were sent to the health services for their clinical evaluation after serological diagnosis. Asymptomatic patients were found in 30.30% (10/33), with some degree of heart disease 18.18% (6/33), in 51.51% (17/33) the clinical evaluation was not performed (S2 Table).

In some samples in which the serological tests showed discrepancies in the positivity results, the PCR verification of the presence of kDNA confirmed infection. Thus, this technique should be incorporated in the diagnostic confirmation of *T*. *cruzi* infection.

Surveys carried out, both in urban and rural areas showed that among the evaluated patients 53% were male, including 6 children below 15 years of age. With reference to socioeconomic data, it was evident that 100% of the rural patients knew about the transmitting insects of Chagas disease and, based on the level and quality of the dwelling, had had repeated contact with these insects. An outstanding finding was the fact that up to four seropositive patients living in the same family unit were reported in the Chararé community (Table 1). This contrasts with the urban patients, of whom everyone knew both, the disease and the insect vector, but most of whom did not remember direct contact with the insect. The diagnosed urban patients reported being the only infected individuals in their family units after all their family members had been assessed. In all cases, regular periodic stays and overnight stays had been reported in rural areas, respectively, areas with high *Atallea* palm density.

The rural community of Chararé is a community with marked poverty and without easy land access, having scarce health resources, and living on subsistence economy based on agriculture, with 56.52% working on their own or in domestic work, and only 30% are salaried. A high percentage, 95.65% of those surveyed had been living in the community for more than 5 years, living with a large variety of domestic/wild animals and frequently exposed to the presence of triatomine vectors. Most of its inhabitants (82.60%) had received basic education (primary and secondary), while 21.73% had no education, being unaware of many aspects of the symptomatology, pathology, treatment or control of the disease (Table 1).

The significant levels of seropositivity found in this study should be considered by the country's health authorities in order to implement better surveillance, prevention, and control programs for this parasitosis.

As for the urban population, 65% had received basic education, and 35% had attended higher education or university, thus having a better understanding of the relevant aspects of Chagas disease. The salaried population is 30%, evidencing in both groups and with high percentage of large families (Table 1).

The clinical manifestations of chagasic infection in Panama are primarily cardiac during both the acute and chronic phases of the disease, with no evidence that the digestive form develops during the later stages of infection [43].

The pattern of Chagas disease in Panama seems to be similar to that of other regions such as Venezuela, Colombia and Central America, where cardiac manifestations prevail [17, 70, 71]. In this sense, and based on the diverse ecobiological conditions existent in Panama, it is likely that, as in other regions of northern South America, DTUs other than TcI are in circulation, both in vectors and in reservoirs. The ecoepidemiological conditions, not fully known in this region, could explain the existence of different DTUs. The import of infected animals or the arrival of foreign vectors is much more complex since the area is remote and difficult to access. A study in Colombia [72] detected the presence of esophageal achalasia associated with Chagas disease, induced by TcI, present in patients diagnosed with this pathology. On the other hand, in Colombia, chagasic patients with mixed cardiac and gastrointestinal involvement were reported, and the genotyping of the tissue samples showed both, TcI in the affected esophagus and TcII in the heart samples [73]. Similarly, mixed infections in insects involving different DTUs (DTU II, III and IV) have been reported [38]. Although in Colombia, DTU I has been reported in most patients, insect vectors and reservoirs, the existence of TcII strains has also been reported in chronic chagasic patients [73]. Likewise, in Venezuela, in ecotopes similar to those evaluated in Panama, human infections caused by TcI and TcII strains and mixed infections in cardiac tissue have been detected with DTUs I, II, III and IV strains [38, 74].

Similar studies suggest that genetically related populations of *T*. *cruzi* possibly exist in the same or adjacent geographic areas and could be involved in determining the development of the same clinical form of the disease. In a study carried out by Vago et al. (2000), multiclonal strains in the heart and esophagus were found, evidencing the first data of possible human differential tissue tropism [75]. Among the three patients with mixed infections (cases 31, 33 and 36) S2 Table, all were residents of Chepo, Las Margaritas and two of them (cases 31 and 36) had cardiac manifestations, while the remainder (case 33) was asymptomatic. As the latter patient was only 14 years old when tested, it cannot be ruled out that, if not treated, she would have developed cardiac disease. On the other hand, taking into consideration the histotropism hypothesis (48), we cannot discard that the other patients studied harbor non-Tc I genotypes that could not be detected in peripheral blood in the samples taken. Follow-up of patients residing in this locality, and especially patients belonging to the families exhibiting mixed infections, would be of much interest in order to find out if genotypes other than TcI may emerge at different lifetime periods along the natural history of their chronic infection.

Research carried out in the Gran Chaco, Argentina, revealed mixed infections of human TcV / TcVI and canine TcI / TcVI in rural areas, demonstrating significant associations between TcV with humans and TcVI with dogs. They also found TcIII, TcI and TcV in dogs and for the first time described TcIII in humans in this area, suggesting that there is contact with wild mammals during hunting or that Tc III may be involved in the domestic cycle [76].

On the other hand, experimental studies carried out in dogs with *T*. *cruzi* Y (TcII) or Col (TcI) have shown that infection by *T*. *cruzi* can be caused by different strains of different DTUs that favor variable clinical forms of chronic Chagas disease, so that the immune response and cardiac lesions can be evaluated. The Col (TcI) strain escapes the host's acute immune response, goes unnoticed by peripheral blood mononuclear cells, and therefore parasitizes organs more rapidly. In contrast, strain Y (TcII), the specific immune response begins in the acute phase, which helps control myocardial injury in the early chronic phase [77]. Similarly, experimental studies in mice refer to Chagas disease heterogenicity from diverse aspects such as organ damage, parasite tropism, immune response, infection phase and strain [78–80].

Studies performed by Mining and coworkers [19], using whole genome tiling arrays for comparative genome analysis of cultured parasite stocks, have revealed that there is much more genotypic diversity among *T*. *cruzi* strains than can be understood using traditional typing methods. In particular, within Tc I, their typing results, together with studies based on microsatellite analyses of Tc I strains [81], indicate that that there is substantially more genetic diversity among Tc I strains than there is among other DTUs. Accordingly, further investigation in isolates from Panama would be needed to assert the intra-DTU variability of circulating Tc I genotypes and their possible transmission cycles.

## Conclusions

Our findings in Chararé confirm TcI as the main circulating DTU in chronic Chagas disease patients from Panama and describe, for the first time, minoritary cases of coinfections with parasite populations belonging to non-Tc I genotypes, such as Tc II, Tc V or Tc VI. The presence of chagasic infections with mixed genetic variants of the parasite in rural areas justifies eco-epidemiological and clinical studies in this new endemic area for Chagas disease in Panama. These investigations should aim to describe the eco-epidemiological implications of the circulation of these DTUs that coinfect together with Tc1 this region of the country, either as a result of the entry of vectors, wild or domestic mammalian reservoirs, or through human migration in this region framed in the natural passage between South America and Central America. It is also a pending task to determine if these new genetic variants of *T*. *cruzi* detected are native.

## Supporting information

S1 Fig Flow-chart of PCR procedures for DTU genotyping in blood samples from chronic Chagas disease patients. Agarose Gels showing amplicons obtained by SL-IR (a), SL-IR I and SL-IR II (b), 24sα rDNA Heminested (c) and A10 fragment Heminested (d) based PCR assays.
(TIF)

S1 Table PCR-based algorithm for Typing DTUs in clinical samples. Primer sequences and amplification conditions.
(DOC)

S2 Table Characterization of *T*. *cruzi* in blood samples from chronic patients with Chagas disease from Panama. HN +: DTU detection by hemi-nested PCR. * HN +: DTU detection by hemi-nested PCR. Untyped: samples that gave nondetectable findings using the DTU typing algorithm.
(DOCX)

S1 Questionnaire (DOCX)

S1 File (DOCX)

S2 File (DOCX)

S3 File (DOCX)

S4 File (DOC)

S5 File (DOCX)
