# Correction: Mixed infections by different *Trypanosoma cruzi* discrete typing units among Chagas disease patients in an endemic community in Panama

**DOI:** 10.1371/journal.pone.0250184

**Published:** 2021-04-08

**Authors:** Alexa Prescilla-Ledezma, Roberto Blandon, Alejandro G. Schijman, Alejandro Benatar, Azael Saldaña, Antonio Osuna

The first author’s name is spelled incorrectly. The correct name is: Alexa Prescilla-Ledezma. The correct citation is: Prescilla-Ledezma A, Blandon R, Schijman AG, Benatar A, Saldaña A, Osuna A (2020) Mixed infections by different *Trypanosoma cruzi* discrete typing units among Chagas disease patients in an endemic community in Panama. PLoS ONE 15(11): e0241921. https://doi.org/10.1371/journal.pone.0241921

The fifth author, Azael Saldaña, should also be noted as a corresponding author. Azael Saldaña’s email address is: asaldana@gorgas.gob.pa.
