# Supplementary figures and images for: Mixed infections by different Trypanosoma cruzi discrete typing units among Chagas disease patients in an endemic community in Panama
Source: PLoS One. 2020 Nov 12;15(11):e0241921. doi: 10.1371/journal.pone.0241921 (PMC7660484; doi:10.1371/journal.pone.0241921)

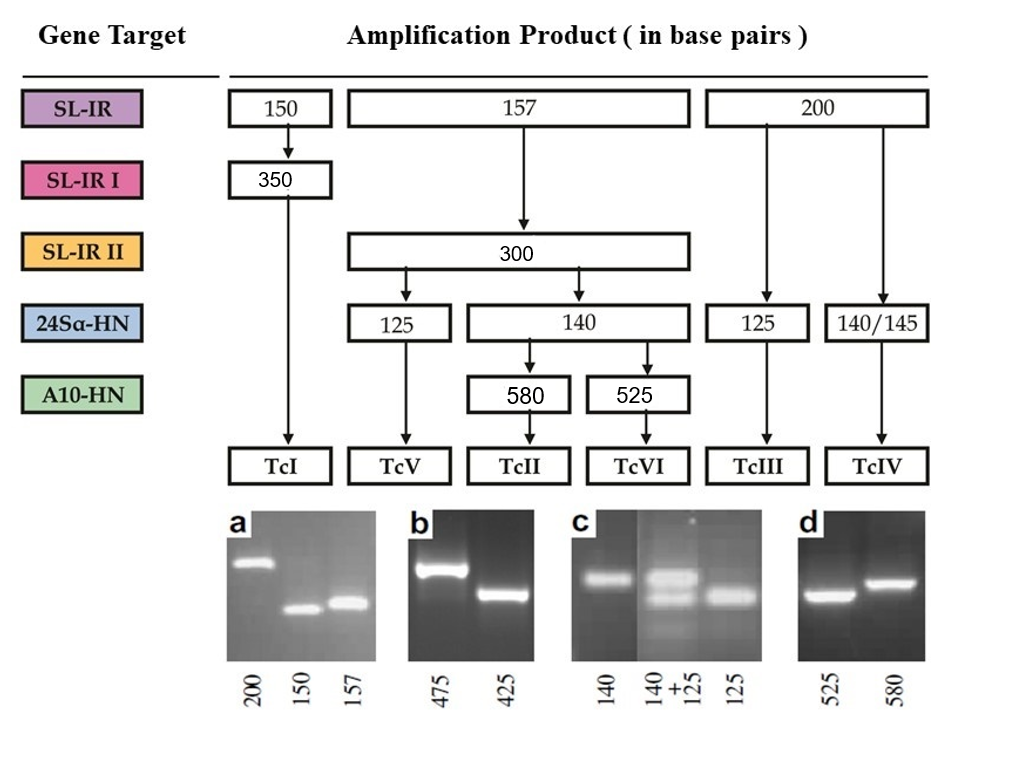

Supplement: S1 Fig — Agarose Gels showing amplicons obtained by SL-IR (a), SL-IR I and SL-IR II (b), 24sα rDNA Heminested (c) and A10 fragment Heminested (d) based PCR assays. (TIF) [file pone.0241921.s001.tif]
