# Supplementary material for: Mixed infections by different Trypanosoma cruzi discrete typing units among Chagas disease patients in an endemic community in Panama
Source: PLoS One. 2020 Nov 12;15(11):e0241921. doi: 10.1371/journal.pone.0241921 (PMC7660484; doi:10.1371/journal.pone.0241921)
Supplement: S1 Table — Primer sequences and amplification conditions. (DOC) [file pone.0241921.s002.doc]

**S1 Table.** PCR-based algorithm for Typing DTUs in clinical samples. Primer sequences and amplification conditions.

D: Denaturation step; A: Annealing step; E: Elongation step; C: Number of Cycles; MT: Melting temperature:
