# Supplementary material for: Mixed infections by different Trypanosoma cruzi discrete typing units among Chagas disease patients in an endemic community in Panama
Source: PLoS One. 2020 Nov 12;15(11):e0241921. doi: 10.1371/journal.pone.0241921 (PMC7660484; doi:10.1371/journal.pone.0241921)
Supplement: S2 Table — HN +: DTU detection by hemi-nested PCR. * HN +: DTU detection by hemi-nested PCR. Untyped: samples that gave nondetectable findings using the DTU typing algorithm. (DOCX) [file pone.0241921.s003.docx]

S2 Table. Characterization of *T. cruzi* in blood samples from chronic patients with Chagas disease from Panama. HN +: DTU detection by hemi-Nested PCR.

A: Rural patients.

| **Case  Nº** | **Sample ID** | **Age** | **Sex** | **Locality** | **Clinical  Profile** | **Western  Blot** | **ELISA** | **Rapid  Test** | **kDNA  -PCR** | **DTUs** |
| --- | --- | --- | --- | --- | --- | --- | --- | --- | --- | --- |
|  |  |  |  |  |  |  |  |  |  |  |
|  |  |  |  |  |  |  |  |  |  |  |
| 1 | 16-16-MCH | 72 | M | Chararé | A | + | - | + | + | Tc I |
| 2 | 17-16-MCH | 35 | F | Chararé | A | + | + | + | - | Untyped |
| 3 | 18-16-MCH | 15 | F | Chararé | A | + | + | + | + | Tc I |
| 4 | 19-16-MCH | 2 | F | Chararé | C | IND | + | + | + | Tc I |
| 5 | 22-16-MCH | 65 | F | Chararé | A | + | - | + | + | Tc I |
| 6 | 24-16-MCH | 76 | F | Chararé | A | IND | - | + | + | Tc I |
| 7 | 25-16-MCH | 77 | M | Chararé | A | + | - | + | + | Tc I HN+* |
| 8 | 26-16-MCH | 35 | M | Chararé | A | + | + | + | + | Tc I |
| 9 | 27-16-MCH | 39 | F | Chararé | A | + | + | + | + | Tc I |
| 10 | 28-16-MCH | 52 | F | Chararé | A | + | + | + | + | Tc I |
| 11 | 29-16-MCH | 6 | F | Chararé | A | IND | - | + | + | Tc I |
| 12 | 31-16-MCH | 51 | M | Chararé | A | + | - | + | + | Tc I |
| 13 | 32-16-MCH | 43 | M | Chararé | A | + | - | + | + | Tc I |
| 14 | 33-16-MCH | 56 | M | Chararé | C | + | + | + | + | Tc I |
| 15 | 34-16-MCH | 12 | M | Chararé | A | + | + | + | + | Tc I |
| 16 | 35-16-MCH | 9 | F | Chararé | A | + | - | + | + | Untyped |
| 17 | 36-16-MCH | 32 | M | Chararé | A | IND | - | + | + | Tc I |
| 18 | 37-16-MCH | 75 | M | Chararé | A | IND | IND | + | + | Tc I |
| 19 | 40-16-MCH | 79 | F | Chararé | A | + | - | + | + | Tc I |
| 20 | 45-16-MCH | 17 | F | Chararé | A | + | + | + | + | Tc I |
| 21 | 46-16-MCH | 34 | F | Chararé | A | + | + | - | + | Tc I |
| 22 | 52-16-MCH | 40 | M | Chararé | C | + | + | + | + | Tc I + Tc II/V/VI |
| 23 | 54-16-MCH | 14 | F | Chararé | A | + | - | + | + | Tc I + Tc II/V/VI |
| 24 | 55-16-MCH | 17 | F | Chararé | A | + | - | + | + | Tc I |
| 25 | 56-16-MCH | 41 | M | Chararé | C | + | + | - | + | Untyped |
| 26 | 62-16-MCH | 39 | F | Chararé | C | + | + | - | + | Tc I + Tc II/V/VI |
| 27 | 64-16-MCH | 54 | M | Chararé | C | + | - | + | + | Tc I |
| 28 | 66-16-MCH | 36 | F | Chararé | A | + | + | + | + | Tc I |
| 29 | 71-16-MCH | 20 | M | Chararé | A | + | + | - | + | Untyped |
| 30 | 72-16-MCH | 22 | M | Chararé | A | + | - | + | + | Untyped |
| 31 | 77-16-MCH | 49 | F | Chararé | A | + | - | + | + | Tc I |
| 32 | 82-16-MCH | 29 | F | Chararé | A | + | - | + | + | Untyped |
| 33 | 91-16-MCH | 57 | M | Chararé | A | + | IND | + | + | Untyped |

B: Urban patients.

| **Case  Nº** | **Sample ID** | **Age** | **Sex** | **Locality** | **Clinical  Profile** | **Western  Blot** | **ELISA** | **Rapid  Test** | **kDNA -PCR** | **DTUs** |
| --- | --- | --- | --- | --- | --- | --- | --- | --- | --- | --- |
|  |  |  |  |  |  |  |  |  |  |  |
|  |  |  |  |  |  |  |  |  |  |  |
| 1 | 02-16-MCH | 49 | F | Arraijan-HST | A | + | + | + | + | Tc I |
| 2 | 03-16-MCH | 41 | M | Chepo-HST | A | + | + | + | + | Untyped |
| 3 | 06-16-MCH | 46 | M | Santiago, Veraguas-HST | C | + | - | + | + | Tc I |
| 4 | 07-16-MCH | 28 | F | Chilibre-HST | A | + | + | - | + | Tc I |
| 5 | 09-16-MCH | 35 | F | Arraijan-CHDr.AAM | A | + | + | - | + | Tc I |
| 6 | 10-16-MCH | 27 | M | Río Abajo-HST | C | + | + | + | + | Tc I |
| 7 | 12-16-MCH | 33 | F | Chepo-HST | C | + | + | + | + | Tc I |
| 8 | 14-16-MCH | 75 | M | Capira-HST | C | + | + | + | + | Tc I |
| 9 | 15-16-MCH | 28 | F | Capira-HST | C | + | + | + | + | Tc I |
| 10 | 53-16-MCH | 46 | M | Las Margaritas-HST | C | + | + | + | + | Tc I |
| 11 | 85-16-MCH | 39 | M | Chepo-HST | A | + | + | + | - | Untyped |
| 12 | 96-16-MCH | 40 | M | Arraijan-HST | C | + | IND | + | + | Tc I |
| 13 | 97-16-MCH | 53 | F | Chilibre-HST | A | + | - | + | + | Untyped |
| 14 | 98-16-MCH | 75 | F | Chilibre-HST | C | IND | - | + | + | Tc I |
| 15 | 99-16-MCH | 39 | M | Alcalde Díaz-CHDr.AAM | A | + | + | + | + | Tc I |
| 16 | 101-16-MCH | 32 | M | Pacora-CHDr.AAM | C | IND | + | + | + | Untyped |
| 17 | 102-16-MCH | 44 | F | Chame-CHDr.AAM | A | + | + | - | + | Tc I |
| 18 | 104-17-MCH | 46 | M | La Chorrera-HEPOTH | A | + | + | - | + | Tc I |
| 19 | 105-17-MCH | 37 | M | La Chorrera-CHDr.AAM | A | + | + | - | + | Untyped |
| 20 | 106-17-MCH | 43 | M | La Chorrera-CHDr.AAM | A | + | - | + | + | Tc I |

* A: Asymptomatic, C : Cardiac. The age is given in years.

* Santo Tomás Hospital (HST), Dr. Arnulfo Arias Madrid Hospital Complex (CHDr.AAM),

Omar Torrijos Herrera Hospital for Pediatric Specialties (HEPOTH)
