# Supplementary material for: Mixed infections by different Trypanosoma cruzi discrete typing units among Chagas disease patients in an endemic community in Panama
Source: PLoS One. 2020 Nov 12;15(11):e0241921. doi: 10.1371/journal.pone.0241921 (PMC7660484; doi:10.1371/journal.pone.0241921)
Supplement: S1 Questionnaire — (DOCX) [file pone.0241921.s004.docx]

**University of Panama**

**Faculty of Medicine**

**Department of Human Microbiology**

**Survey on the Knowledge, Attitudes and Practices concerning Chagas Disease**

**Code of survey:_______________________**

**Number of questionnaire:________________**

**Name of community:___________________**

The Department of Human Microbiology of the Faculty of Medicine of University of Panama is carrying out a survey to determine the knowledge, attitudes and practices concerning Chagas disease. The information contained in this form, which will allow the identification of any individual, will be gathered with the guarantee that it will be kept strictly confidential and will be used only for the purposes established in this study.

***1***. General Data

| 1. **Sex** | | | | | | Female……..… | | | | | | | | |  | | 1 |  | **2. How old are you (years)?** ………………… | | | | | | | | | |  |  | |
| --- | --- | --- | --- | --- | --- | --- | --- | --- | --- | --- | --- | --- | --- | --- | --- | --- | --- | --- | --- | --- | --- | --- | --- | --- | --- | --- | --- | --- | --- | --- | --- |
|  | | | | | | Male………..… | | | | | | | | |  | | 2 |  |  | | | | |  | | | |  | | | |
|  | | |  | | | |  | | | | | | | | | | |  |  | | | | |  | | | |  | | | |
| 3. **Which degree or how many years of schooling did you complete?** | | | | | | | | | | | | | | | | | |  | 4**. Do you know how to read and write?** | | | | | | | | | | | | |
| None ………………………… | | | | | | | |  | | 1 | | | | | | | |  | Yes…………. | | | | |  | 1 | | | | | | |
| Primary ……………………… | | | | | | | |  | | 2 | | | |  | | | |  | No..………… | | | | |  | 2 | | | | | | |
| Secondary…………………… | | | | | | | |  | | 3 | | | | Move on to question 5 | | | |  |  | | | | |  | | | |  | | | |
| Vocational ............................ | | | | | | | |  | | 4 | | | |  |  |  |  |  |  | | | | |  | | | |  | | | |
| Higher education, not university | | | | | | | |  | | 5 | | | |  |  |  |  |  |  | | | | |  | | | |  | | | |
| Higher education, University … | | | | | | | |  | | 66 | | | |  |  |  |  |  |  | | | | |  | | | |  | | | |
|  | | | | | | | |  | |  | | | | | | | |  |  | | | | |  | | | |  | | | |
| 5. **Which occupational category did you belong to in the last month?** | | | | | | | | | | | | | | | | | |  | **5a. What is your current weekly income?** | | | | | | | | | | | | |
| Employed or salaried…….....… | | | | | | | | | | | |  | 1 | | | | |  | Less than B/US $ 50,00……………… | | | | | | |  | | 1 | | | |
| Self-employed…………………. | | | | | | | | | | | |  | 2 | | | | |  | Between B/US $ 51,00 and B/US $ 100,00 | | | | | | |  | | 2 | | | |
| Unpaid family helper ……..…… | | | | | | | | | | | |  |  | | | | |  | Between B./US $ 101,00 and B/US $ 150,00 | | | | | | |  | | 3 | | | |
|  |  |  |  |  |  |  |  |  |  |  |  |  | 3 | | | Move on to question 6 | |  | More than B/US $ 151,00……………. | | | | | | |  | | 4 | | | |
| Housework……………………… | | | | | | | | | | | |  | 4 | | |  |  |  |  | | | | | | |  | |  | | | |
| Student………………………….. | | | | | | | | | | | |  | 5 | | |  |  |  | 5b. What kind of occupation, profession or job do you have? | | | | | | | | | | | | |
| Unemployed …………………… | | | | | | | | | | | |  | 6 | | |  |  |  |  | |  | | | | | | |  | | | |
|  | | | | | | | | |  | |  | | | | | | |  |  | |  | | | | | | |  | | | |
|  | | | | | | | | | | | | | | | | | |  |  | | | | | | | | | | | | |
| 6. **How long have you been living in this community?** | | | | | | | | | | | | | | | | | |  | 7. **How many people live in your home?** | | | | | | | | | | | | |
|  |  | | | |  | | | | | | | | | | | | |  |  | | |  |  | | | | | | | | |
|  |  | | | |  | | | | | | | | | | | | |  |  | | |  |  | | | | | | | | |
| **8. How many children (below the age of 14) live in the home?** | | | | | | | | | | | | | | | | | |  | 8.a **Provide the name and age of the child/children** | | | | | | | | | | | | |
|  | |  | |  | | | | | | | | | | | | | |  |  | Name | | | | | | | Age | | | | |
|  | | | | | | | | | | | | | | | | | |  |  |  | | | | | | |  | | | | 1 |
|  | | | | | | | | | | | | | | | | | |  |  |  | | | | | | |  | | | | 2 |
|  | | | | | | | | | | | | | | | | | |  |  |  | | | | | | |  | | | | 3 |
|  | | | | | | | | | | | | | | | | | |  |  |  | | | | | | |  | | | | 4 |
|  | | | | | | | | | | | | | | | | | |  |  |  | | | | | | |  | | | | 5 |
|  | | | | | | | | | | | | | | | | | |  |  |  | | | | | | |  | | | | 6 |
|  | | | | | | | | | | | | | | | | | |  |  |  | | | | | | |  | | | | 7 |
|  | | | | | | | | | | | | | | | | | |  |  |  | | | | | | |  | | | | 8 |

***2***. Characteristics of the Dwelling

| 9. **What kind of material is the largest part of the roof made of?** | | | | | | | | | | | |  | | 10. **What kind of material is the largest part of the walls made of?** | | | | | | | | | | | | | | | |
| --- | --- | --- | --- | --- | --- | --- | --- | --- | --- | --- | --- | --- | --- | --- | --- | --- | --- | --- | --- | --- | --- | --- | --- | --- | --- | --- | --- | --- | --- |
| Fronds or straw ………………...… | | | | | |  | 1 | | | | |  | | Slabs, bricks, Stone………… | | | | | | | | |  | 1 | | | | | |
| Metal (zinc, aluminium) …….……. | | | | | |  | 2 | | | | |  | | Wood (boards, logs)………… | | | | | | | | |  | 2 | | | | | |
| Wood……………………………….. | | | | | |  | 3 | | | | |  | | Rushes and mud………….… | | | | | | | | |  | 3 | | | | | |
| Others (specify) | | |  | | | | | | | | |  | | Adobe…………………….….. | | | | | | | | |  | 4 | | | | | |
|  |  | | | |  | | | | | | |  | | Bamboo…………………….… | | | | | | | | |  | 5 | | | | | |
|  |  | | | |  | | | | | | |  | | Others (specify) | | | | | | | |  | | | | | | | |
|  |  | | | |  | | | | | | |  | |  | |  | | | | | | |  | | | | | | |
|  |  | | | |  | | | | | | |  | |  | |  | | | | | | |  | | | | | | |
| **11**. **What kind of material is the largest part of the floor made of?** | | | | | | | | | | | |  | | **12. How many bedrooms does the home have?** | | | | | | | | | | | | | |  | |
|  |  |  |  |  |  |  |  |  |  |  |  |  |  |  |  |  |  |  |  |  |  |  |  |  |  |  |  |  | |
| Wood …………………… | | | |  | 1 | | | | | | |  | |  | |  | | | | | | |  | | | | | | |
| Dirt floor………………… | | | |  | 2 | | | | | | |  | | **13. Does the home have any extensions?** | | | | | | | | | | | | | | | |
| Cement……………….… | | | |  | 3 | | | | | | |  | | Yes…………. | |  | | 1 | | | | | | | | | | | |
| Others (cane, sticks)… | | | |  | | | | | | | |  | | No………….. | |  | | 2 | | | Move on to question 15 | | | | |  | | | |
|  | | | |  |  | | | | | | |  | |  | |  | |  | | | | | | | | | | | |
|  | | | |  |  | | | | | | |  | |  | |  | |  | | | | | | | | | | | |
| 14. **Tell us what kind of extensions your home has and where they are situated?** | | | | | | | | | | | | | | | | | | | | | | | | | | | | | |
| Kitchen……………………………..… | | | | | | | |  | | 1 | | | Away from the home | | | |  | | 1 | Adjacent (less than 10 m away) ) | | | | | | |  | | 2 |
| Storeroom …………………………… | | | | | | | |  | | 2 | | | Away from the home | | | |  | | 1 | Adjacent | | | | | | |  | | 2 |
| Chicken coop………………..……… | | | | | | | |  | | 3 | | | Away from the home | | | |  | | 1 | Adjacent | | | | | | |  | | 2 |
| Pigsty………………………………... | | | | | | | |  | | 4 | | | Away from the home | | | |  | | 1 | Adjacent | | | | | | |  | | 2 |
| Livestock pen………………….……. | | | | | | | |  | | 5 | | | Away from the home | | | |  | | 1 | Adjacent | | | | | | |  | | 2 |
| Bathrooms ……………………….…. | | | | | | | |  | | 6 | | | Away from the home | | | |  | | 1 | Adjacent | | | | | | |  | | 2 |
| Lavatories ………………………….. | | | | | | | |  | | 7 | | | Away from the home | | | |  | | 1 | Adjacent | | | | | | |  | | 2 |
| Firewood stack……………………... | | | | | | | |  | | 8 | | | Away from the home | | | |  | | 1 | Adjacent | | | | | | |  | | 2 |
| Stone stack………………………….. | | | | | | | |  | | 9 | | | Away from the home | | | |  | | 1 | Adjacent | | | | | | |  | | 2 |
| Others (specify) | | |  | | | | | | | | | | Away from the home | | | |  | | 1 | Adjacent | | | | | | |  | | 2 |
|  | | |  | | | | | | | | | |  | | | |  | |  | | |  | | |  | | |  | |
|  | | |  | | | | | | | | | |  | | | |  | |  | | |  | | |  | | |  | |
| **15. What kind of lighting do you use in the dwelling?** | | | | | | | | | | | | |  | |  | | | | | | | | | | | | | | |
| Velas………….……………………….. | | | | | | | | |  | | 1 | |  | |  | | | | | | |  | | |  | | |  | |
| Kerosene lamps…………………….... | | | | | | | | |  | | 2 | |  | |  | | | | | | |  | | |  | | |  | |
| Battery-powered lamps……………… | | | | | | | | |  | | 3 | |  | |  | | | | | | |  | | |  | | |  | |
| Electric light……………………….…... | | | | | | | | |  | | 4 | |  | |  | | | | | | |  | | |  | | |  | |
| Others (specify) : | |  | | | | | | | | | | |  | |  | | | | | | |  | | |  | | |  | |

***3***. Presence of Animals

| **16. Which of the following animals do you own?** | | | | | | | | | | | | | | | | | | | | | | | | | | | | | | | | |
| --- | --- | --- | --- | --- | --- | --- | --- | --- | --- | --- | --- | --- | --- | --- | --- | --- | --- | --- | --- | --- | --- | --- | --- | --- | --- | --- | --- | --- | --- | --- | --- | --- |
| Chickens………….… | | |  | | | 1 | | | | | | |  | Do they sleep in the home? | | | | | Yes | | | |  | | 1 | | | No | | 2 | | 2 |
| Dogs………………… | | |  | | | 2 | | | | | | |  | Do they sleep in the home? | | | | | Yes | | | |  | | 1 | | | No | |  | | 2 |
| Cats…………………. | | |  | | | 3 | | | | | | |  | Do they sleep in the home? | | | | | Yes | | | |  | | 1 | | | No | |  | | 2 |
| Pigs…………………. | | |  | | | 4 | | | | | | |  | Do they sleep in the home? | | | | | Yes | | | |  | | 1 | | | No | |  | | 2 |
| Horses……………… | | |  | | | 5 | | | | | | |  | Do they sleep in the home? | | | | | Yes | | | |  | | 1 | | | No | |  | | 2 |
| Ducks ………………. | | |  | | | 6 | | | | | | |  | Do they sleep in the home? | | | | | Yes | | | |  | | 1 | | | No | |  | | 2 |
| Rabbits……………… | | |  | | | 7 | | | | | | |  | Do they sleep in the home? | | | | | Yes | | | |  | | 1 | | | No | |  | | 2 |
| Others (which?) |  | | | | | | | | | | | |  | Do they sleep in the home? | | | | | Yes | | | |  | | 1 | | | No | |  | | 2 |
| I don’t own any | | |  | | | 8 Move on to question 19 | | | | | | | | | |  | | | | | | | | | | | | | | | | |
|  | | | | | | | | | | | | | | | | | | | | | | | | | | | | | | | | |
| 17. **Do you keep any of these animals in livestock pens or cages?** | | | | | | | | | | | | | | | | |  | 18. **At what distance are the caged or penned animals kept?** | | | | | | | | | | | | | | |
| Yes………….. | |  | | 1 | | | | | | | | | | | | |  | Distant………………. | |  | | | 1 | | | | | | | | | |
| No…………… | |  | | 2 | | | | Move on to question 19 | | | | | | |  | |  | Adjacent……………. | |  | | | 2 | | | | | | | | | |
|  | | | | | | | | | | | | | | | | | | | | | | | | | | | | | | | | |
| 19. **Which kind of wild animals do you frequently see near your dwelling?** | | | | | | | | | | | | | | | | |  | 20. **Which animals have you seen in/beneath the royal palms?** | | | | | | | | | | | | | | |
|  | | | | | | | 1.Yes | | | 2. No | |  | | | | |  |  | | | | | | 1.Yes | | | 2. No | | | |  | |
| Opposums or foxes………. | | | | | | | | |  |  | 1 | | | | | |  | Opposums or foxes………. | | | | | | | |  |  | | 1 | | | |
| Squirrels ……………..…… | | | | | | | | |  |  | 2 | | | | | |  | Squirrels………………….… | | | | | | | |  |  | | 2 | | | |
| Mice……………………..… | | | | | | | | |  |  | 3 | | | | | |  | Mice ………………………… | | | | | | | |  |  | | 3 | | | |
| Monkeys………………..… | | | | | | | | |  |  | 4 | | | | | |  | Monkeys …………………... | | | | | | | |  |  | | 4 | | | |
| Birds/Fowls…………….… | | | | | | | | |  |  | 5 | | | | | |  | Birds/Fowls ……………..… | | | | | | | |  |  | | 5 | | | |
| Sloths …………………….. | | | | | | | | |  |  | 6 | | | | | |  | Sloths………………………. | | | | | | | |  |  | | 6 | | | |
| Porcupine………………… | | | | | | | | |  |  | 7 | | | | | |  | Bats……………………..….. | | | | | | | |  |  | | 7 | | | |
| Bats……………………….. | | | | | | | | |  |  | 8 | | | | | |  | Others (specify): | | | |  | | | | | | | | | | |
| Others (specify): | | | | |  | | | | | | | | | | | |  |  | | |  | | | | | | | | | | | |

***4***. Vegetation

| 21. **What kind of vegetation is near your home (less than 50 meters away)?** | | | | | |  | 22. **How do you use royal palms and their derivatives?** | | | | |
| --- | --- | --- | --- | --- | --- | --- | --- | --- | --- | --- | --- |
| There is no vegetation…….. | |  | | 1 | |  | None……………………………….. | | |  | 1 |
| Royal palms………………… | |  | | 2 | (Move on to question 24) |  | Wine……………………………….. | | |  | 2 |
| Fruit trees…………………… | |  | | 3 |  |  | Shade……………………………... | | |  | 3 |
| Bushes………………………. | |  | | 4 |  |  | Fronds……………………..……… | | |  | 4 |
| Weeds………………………. | |  | | 5 |  |  | Firewood ………………………….. | | |  | 5 |
| Vegetables…………………. | |  | | 6 |  |  | Others (specify) |  | | | |
| Other palm trees…………... | |  | | 7 |  |  |  | | | | |
| Coconut palm trees………… | |  | | 8 |  |  |  | | | | |
| Others (specify) |  | | | | |  | 23. **How far away are the royal palms from your dwellling?** | | | | |
|  | | |  | | |  | Less than 10 meters……….. | |  | | 1 |
|  | | |  | | |  | More than 10 meters……….. | |  | | 2 |

***5***. Knowledge **concerning** Chagas Disease

| **24**. **Have you heard about or do you know what Chagas disease is?** | | | | | | | | | | | | | | | | | |  | | **25. How, or who from, did you learn about Chagas disease?** | | | | | | | | | | | | | | | | | | | | | | | | |
| --- | --- | --- | --- | --- | --- | --- | --- | --- | --- | --- | --- | --- | --- | --- | --- | --- | --- | --- | --- | --- | --- | --- | --- | --- | --- | --- | --- | --- | --- | --- | --- | --- | --- | --- | --- | --- | --- | --- | --- | --- | --- | --- | --- | --- |
| Yes………….…… |  | | 1 | | | | | | | | | | | | | | |  | | School………………………..… | | | | | | | | | | |  | | 1 | | | | | | | | | | | |
| No………………… |  | | 2 Move on to question 34 | | | | | | | | | | | | | | |  | | Radio…………………………… | | | | | | | | | | |  | | 2 | | | | | | | | | | | |
|  |  | |  | | | | | | | | | | | | | | |  | | Newspaper…………………….. | | | | | | | | | | |  | | 3 | | | | | | | | | | | |
|  |  | |  | | | | | | | | | | | | | | |  | | Church or worship…………..… | | | | | | | | | | |  | | 4 | | | | | | | | | | | |
|  |  | |  | | | | | | | | | | | | | | |  | | Health care personnel………… | | | | | | | | | | |  | | 5 | | | | | | | | | | | |
|  |  | |  | | | | | | | | | | | | | | |  | | Health committees…………….. | | | | | | | | | | |  | | 6 | | | | | | | | | | | |
|  |  | |  | | | | | | | | | | | | | | |  | | Others (specify): | | | | | | | |  | | | | | | | | | | | | | | | | |
|  |  | |  | | | | | | | | | | | | | | |  | |  | | | | | | | | | | | | |  | |  | | | | | | | | | |
| **26**. **What kind of measures do you take against Chagas?** | | | | | | | | | | | | | | | | | |  | | **27. How do you think one can be infected by Chagas?** | | | | | | | | | | | | | | | | | | | | | | | | |
|  | | | | | | | | | | | | | 1. Yes | | | 2. No | |  | |  | | | | | | | | | | 1.Yes | | | | | | | 2. No | | | | |  | | |
| Keeping the home tidy…………….… | | | | | | | | | | | | | |  | |  | 1 | |  | Being bitten by the bug…………. | | | | | | | | | | | | | |  | | |  | | | 1 | | | | |
| Spraying insecticides………………... | | | | | | | | | | | | | |  | |  | 2 | |  | Person-to-person……………….. | | | | | | | | | | | | | |  | | |  | | | 2 | | | | |
| Preventing animals from entering the dwelling ………………….……………. | | | | | | | | | | | | | |  | |  |  | |  | Contaminated foodstuffs | | | | | | | | | | | | | |  | | |  | | | 3 | | | | |
|  |  |  |  |  |  |  |  |  |  |  |  |  |  |  | |  | 3 | |  |  |  |  |  |  |  |  |  |  |  |  |  |  |  |  |  |  |  |  |  |  |  |  |  |  |
| Keeping windows and doors closed at night………………………………… | | | | | | | | | | | | | |  | |  |  | |  | Contamination with the bug’s excrements ………………….. | | | | | | | | | | | | | |  | | |  | | | 4 | | | | |
|  |  |  |  |  |  |  |  |  |  |  |  |  |  |  | |  | 4 | |  | Don’t know………………………… | | | | | | | | | | | | | |  | | |  | | | 5 | | | | |
| Keeping away from royal palms near the home………………………………. | | | | | | | | | | | | | |  | |  |  | |  | Others (specify) | | | | | |  | | | | | | | | | | | | | | | | | | |
|  |  |  |  |  |  |  |  |  |  |  |  |  |  |  | |  | 5 | |  |  | | | | | |  | | | | | | | | | | | | | | | | | | |
| Avoiding untidiness near the home………………….………….. | | | | | | | | | | | | | |  | |  |  | |  |  | | | | | |  | | | | | | | | | | | | | | | | | | |
|  |  |  |  |  |  |  |  |  |  |  |  |  |  |  | |  | 6 | |  |  | | | | | |  | | | | | | | | | | | | | | | | | | |
| Periodically checking for the presence of bugs in the dwelling. | | | | | | | | | | | | | |  | |  |  | |  |  | | | | | |  | | | | | | | | | | | | | | | | | | |
|  |  |  |  |  |  |  |  |  |  |  |  |  |  |  | |  | 7 | |  |  |  |  |  |  |  |  |  |  |  |  |  |  |  |  |  |  |  |  |  |  |  |  |  |  |
|  | | |  | | |  | | | | | | | | | | | |  | |  | | |  | | | | | | | | |  | | | | | | | | | | | | |
| **28**. **Have you ever had a medical examination for the diagnosis of Chagas disease?** | | | | | | | | | | | | | | | | | |  | | **29**. **Do you have Chagas disease?** | | | | | | | | | | | | | | | | | | | | | | | | |
| Yes…………… | | |  | | 1 | | | | | | | | | | | | |  | | Yes…..…….… | |  | | 1 | | | | | | | | | | | | | | | | | | | | |
| No………….… | | |  | | 2 | | | | | | | | | | | | |  | | No…………….. | |  | | 2 | | | | | | | | | | | | | | | | | | | | |
| Don’t know …. | | |  | | 3 | | | | | | | | | | | | |  | | Don’t know…… | |  | | 3 | | | | | | | | | | | | | | | | | | | | |
|  | | |  | | |  | | | | | | | | | | | |  | |  | | | | | |  | | |  | | | | | | | | | | | | | | | |
| **30**. **Do you know the symptoms of people who have Chagas disease?** | | | | | | | | | | | | | | | | | |  | | **31**. **What are the symptoms?** | | | | | | | | | | | | | | | | | | | | | | | | |
| Yes…………… | |  | | 1 | | | | | | | | | | | | | |  | | Fever……………………..……… | | | | | | | | | | | | |  | | | | | 1 | | | | | | |
| No……………. | |  | | 2 Move on to question 32 | | | | | | | | | | | | | |  | | Headache………………………… | | | | | | | | | | | | |  | | | | | 2 | | | | | | |
|  | | | | | | | | | | |  | |  | | | | |  | | Swollen eyelids……………….… | | | | | | | | | | | | |  | | | | | 3 | | | | | | |
|  |  |  |  |  |  |  |  |  |  |  |  |  |  |  |  |  |  |  |  |  |  |  |  |  |  |  |  |  |  |  |  |  |  | | | | |  |  |  |  |  |  |  |
|  | | | | | | | | | | |  | |  | | | | |  | | Heart disease………….……….. | | | | | | | | | | | | |  | | | | | 4 | | | | | | |
|  | | | | | | | | | | |  | |  | | | | |  | | Malaise…………………………… | | | | | | | | | | | | |  | | | | | 5 | | | | | | |
|  | | | | | | | | | | |  | |  | | | | |  | | Inflammation at the point of the bite……………………………. | | | | | | | | | | | | |  | | | | |  | | | | | | |
|  |  |  |  |  |  |  |  |  |  |  |  |  |  |  |  |  |  |  |  |  |  |  |  |  |  |  |  |  |  |  |  |  |  | | | | | 6 | | | | | | |
|  | | | | | | | | | | |  | |  | | | | |  | | Others (specify) | | | | | |  | | | | | | | | | | | | | | | | | | |
|  | | | | | | | | | | |  | |  | | | | |  | |  | | |  | | | | | | | | |  | | | | | | | | | | | | |
| **32**. **Do you know that the disease requires medical treatment?** | | | | | | | | | | | | | | | | | |  | | **33**. **Are you being treated for Chagas disease?** | | | | | | | | | | | | | | | | | | | | | | | | |
| Yes……………..……… | | | | | | |  | | 1 | | | | | | | | |  | | Yes……..…….. | | | | |  | | 1 | | | | | | | | | | | | | | | | | |
| No……………………… | | | | | | |  | | 2 | | | Move on to question 34 | | | | | |  | | No……...……… | | | | |  | | 2 | | | | | | | | | | | | | | | | |  |
|  | | | | | | | |  | |  | | | | | | | |  | |  | | | | | | | | | | | | | | | |  | | | | |  | | | |
|  | |  | |  | | | | | | | | | | | | | |  | |  | | | | | | | | | | | | | | | |  | | | | |  | | | |
| **34**. **Do you know that the disease can be very serious and sometimes fatal?** | | | | | | | | | | | | | | | | | |  | | **35. Is any member of your community:** | | | | | | | | | | | | | | | | | | | | | | | | |
| Yes…………….… | |  | | 1 | | | | | | | | | | | | | |  | | ill with Chagas?.......................... | | | | | | | | | | | | | | | |  | | | 1 | | | | | |
| No………………... | |  | | 2 | | | | | | | | | | | | | |  | | Has died of Chagas? ................. | | | | | | | | | | | | | | | |  | | | 2 | | | | | |
|  | | | | | | | | | | |  | |  | | | | |  | | There have been no cases………. | | | | | | | | | | | | | | | |  | | | 3 | | | | | |
|  | | | | | | | | | | | | | | | | | |  | | |  | | | | | | | | | | | | | | |  | | | | | | |  | |
| **36**. **Is anybody in your dwelling?** | | | | | | | | | | | | | | | | | How many? | | | | Kinship | | | | | | | | | | | | | | | | | | | | | |  | |
| ill with Chagas?................................. | | | | | | | | | | | | | |  | 1 | | |  | |  |  | | | | | | | | | | | | | | | | | | | | | |  | |
| Being treated?.................................. | | | | | | | | | | | | | |  | 2 | | |  | |  |  | | | | | | | | | | | | | | | | | | | | | |  | |
| Has died of Chagas?........................ | | | | | | | | | | | | | |  | 3 | | |  | |  |  | | | | | | | | | | | | | | | | | | | | | |  | |
| Don’t know........................................ | | | | | | | | | | | | | |  | 4 | | |  | |  |  | | | | | | | | | | | | | | | | | | | | | |  | |

***6***. Knowledge about the Vector of Chagas Disease

| 37. **Are you able to identify the feces of bugs?** | | | | | | | | | | |  | 38. **Do you know that Chagas disease is transmitted through the feces left by the bug at the spot of the bite?** | | | | | | | | | | | | | | | |
| --- | --- | --- | --- | --- | --- | --- | --- | --- | --- | --- | --- | --- | --- | --- | --- | --- | --- | --- | --- | --- | --- | --- | --- | --- | --- | --- | --- |
| Yes………………….. |  | 1 | | | | | | | | |  | Yes…………………………………… | | | | |  | | 1 | | | | | | | | |
| No…………………… |  | 2 | | | | | | | | |  | No………………………………....… | | | | |  | | 2 | | | | | | | | |
| **39**. **Which of these do you recognize?** | | | | | | | | | | |  | **40a**. **Have you received training or information in order to recognize the bugs?** | | | | | | | | | | | | | | | |
| Rhodnius pallescens | | | | | | | | |  | 1 |  | Yes… | |  | | 1 | | | | | | | | | | | |
| Rhodnius pallescens nymphs | | | | | | | | |  | 2 |  | No… | |  | | 2 Move on to question 44 | | | | | | | | | | | |
| Triatoma dimidiata | | | | | | | | |  | 3 |  |  | |  | |  | | | | | | | | | | | |
| Triatoma dimidiata nymphs | | | | | | | | |  | 4 |  | **40b. Who from?** | |  | | | | | | | | | | | | | |
| None | | | | | | | | |  | 5 |  |  |  | | | | | | | | | | | | | | |
|  | | | | | | |  | |  | |  |  | |  | |  | | | | | | | | | | | |
| **41.** **Where have you seen the bugs?** | | | | | | | | | | |  | **42.** **At what time did you see them?** | | | | | | | | | | | | | | | |
| In the forest……….....…… | | | |  | 1 | | | | | |  | In the morning………………………….. | | | | | | | | | |  | | **1** | | | |
| Inside the home………….. | | | |  | 2 | | | | | |  | In the afternoon/evening……………… | | | | | | | | | |  | | **2** | | | |
| Around the home………… | | | |  | 3 | | | | | |  | At night…………………………………. | | | | | | | | | |  | | **3** | | | |
| At school…………………. | | | |  | 4 | | | | | |  | At any time……………………………… | | | | | | | | | |  | | **4** | | | |
| In some nests…………….. | | | |  | 5 | | | | | |  |  | | | | | | | | |  | |  | | | | |
| I have never seen them.. | | | |  | 6 | | Move on to question 44 | | | |  |  | | | | | | | | |  | |  | | | | |
|  | | | |  |  | | | | | |  |  | | | | | | | | |  | |  | | | | |
| **43. Do you know where these bugs live?** | | | | | | | | | | |  | **44. Do you know what the bugs feed on?** | | | | | | | | | | | | | | | |
|  | | | | | | 1. Yes | | 2. No |  | |  | Fruit… | | | | | |  | |  | | | | | | | |
|  |  |  |  |  |  |  |  |  |  |  |  |  |  |  |  |  |  |  | | 1 | | | | | | | |
| In the forest ………………..… | | | | | |  | |  | 1 | |  | Plants…… | | | | | |  | | 2 | | | | | | | |
| On royal palms……………….. | | | | | |  | |  | 2 | |  | Blood | | | | | |  | | 3 | | | | | | | |
| On the ground……………..…. | | | | | |  | |  | 3 | |  | Don’t know | | | | | |  | | 4 | | | | | | | |
| In lairs or nests………………. | | | | | |  | |  | 4 | |  | Others(specify) | | | | | | |  | | | | | | | | |
| In between logs…………….... | | | | | |  | |  | 5 | |  |  | | | | | | | | | | | |  | | | |
| Inside the home……………... | | | | | |  | |  | 6 | |  |  | | | | | | | | | | | | | | | |
| In the kitchen…………………. | | | | | |  | |  | 7 | |  |  |  |  |  |  |  |  |  |  |  |  |  |  |  |  |  |
| Cracks of the walls………….. | | | | | |  | |  | 8 | |  |  |  |  |  |  |  |  |  |  |  |  |  |  |  |  |  |
| Don’t know…………………… | | | | | |  | |  | 9 | |  |  | | |  | |  | | | | | | | | | | |
| Below stones…………………. | | | | | |  | |  | 10 | |  |  | | |  | |  | | | | | | | | | | |
| Others (specify) | | |  | | | | | | | |  |  | | |  | |  | | | | | | | | | | |
|  | | | | | |  | |  | | |  |  | | | | | | | | | | |  | | |  | |
| 45. **Have you ever been bitten by these bugs?** | | | | | | | | | | |  | 46. **Has any of your family members ever been bitten by these bugs?** | | | | | | | | | | | | | | | |
| Yes…………… |  | 1 | | | | | | | | |  | Yes……………………………..… | | | | | |  | | 1 | | | | | | | |
| No……………. |  | 2 | | | | | | | | |  | No………………………………… | | | | | |  | | 2 | | | | | | | |
|  |  |  | | | | | | | | |  | Don’t know …………………….... | | | | | |  | | 3 | | | | | | | |
|  |  |  | | | | | | | | |  |  | | | | | | | | | | | | |  | |  |
| 47. **Do you know that the bugs are attracted to the dwelling by domesticated animals and rats?** | | | | | | | | | | |  |  | | | | | | | | | | | | | | | |
| Yes………….. |  | 1 | | | | | | | | |  |  | | | |  | |  | | | | | | | | | |
| No…………… |  | 2 | | | | | | | | |  |  | | | |  | |  | | | | | | | | | |

***7***. Practices

| **48. Do you use a mosquito net when you sleep?** | | | | | | | | |  | **49. Who uses a mosquito net in your dwelling?** | | | | | | | | | | | | | | | | |
| --- | --- | --- | --- | --- | --- | --- | --- | --- | --- | --- | --- | --- | --- | --- | --- | --- | --- | --- | --- | --- | --- | --- | --- | --- | --- | --- |
| Yes……………….….. | |  | | 1 | | | | |  | Adults……………….… | | | | |  | | 1 | | | | | | | | | |
| No…………………… | |  | | 2 | | | | |  | Children………….…… | | | | |  | | 2 | | | | | | | | | |
| I don’t use one………. | |  | | 3 | | | | |  | Both…………………... | | | | |  | | 3 | | | | | | | | | |
|  | |  | |  | | | | |  | Nobody………………….. | | | | |  | | 4 | | | | | | | | | |
|  | | | | | | | | |  |  | | | | | | | | | | | | | | | | |
| **50. Do you use a repellent, lotion or citronella incense sticks?** | | | | | | | | |  | **51. Do you use metal mosquito nets in the windows and doors?** | | | | | | | | | | | | | | | | |
| Always…………………... | |  | | 1 | | | | |  | Yes………………….…. | | | | |  | | | 1 | | | | | | | | |
| Sometimes……………... | |  | | 2 | | | | |  | No……………………… | | | | |  | | | 2 | | | | | | | | |
| I don’t use any……….… | |  | | 3 | | | | |  |  | | | | |  | | |  | | | | | | | | |
|  | |  | |  | | | | |  |  | | | | |  | | |  | | | | | | | | |
| **52.** **How often do you clean and tidy inside the dwelling?** | | | | | | | | |  | **53. How often do you clean and tidy the area surrounding your dwelling**? | | | | | | | | | | | | | | | | |
| Every day…………… | | | | |  | 1 | | |  | Every day…………….…… | | | | | | | | |  | 1 | | | | | | |
| Once a week………. | | | | |  | 2 | | |  | Once a week…….……….. | | | | | | | | |  | 2 | | | | | | |
| Once a month…..…. | | | | |  | 3 | | |  | Once a month…………….. | | | | | | | | |  | 3 | | | | | | |
| Never……………….. | | | | |  | 4 | | |  | Never ………………….….. | | | | | | | | |  | 4 | | | | | | |
| Other…………….…. | | | | |  | | | |  | Other……………... | | | | | |  | | | | | | | | | | |
|  | | | | |  |  | | |  |  | | | | | | | | |  |  | | | | | | |
| **54.** **Do you tend to stick objects (calendars or stickers) on the walls of your home or do you cover them otherwise?** | | | | | | | | |  | 55. **Do you store inside the house?** | | | | | | | | | | | | | | | | |
| Yes………..…………. | | | | |  | 1 | | |  | Firewood…… | | | | | | | | |  | 1 | | | | | | |
| No…………….……… | | | | |  | 2 | | |  | Farm produce in the home? | | | | | | | | |  | 2 | | | | | | |
|  | | | | |  |  | | |  | I don’t store anything .. | | | | | | | | |  | 3 | | | | | | |
|  | | | | |  |  | | |  |  | | | | | | | | |  |  | | | | | | |
| **56**. **Do you seal holes, cracks or splits of your dwelling?** | | | | | | | | |  | **57. Do you store building material in the dwelling?** | | | | | | | | | | | | | | | | |
| Always……………..…. | | | | |  | 1 | | |  | Yes………… |  | | 1 | | | | | | | | | | | | | |
| Never.………………… | | | | |  | 2 | | |  | No……..…… |  | | 2 | | | | | | | | | | | | | |
|  | | | | |  |  | | |  |  | | | | | | | | |  |  | | | | | | |
| **58**. **In case you find bugs, do you use gloves or any other type of protection to cover your hands to catch them?** | | | | | | | | |  | **59. What do you sleep on?** | | | | | | | | | | | | | | | | |
| Yes………..….… |  | | 1 | | | | | |  | A wooden bed……………..… | | | | | | | | |  | 1 | | | | | | |
| No……..……….. |  | | 2 | | | | | |  | A bed with a mattress………. | | | | | | | | |  | 2 | | | | | | |
| I don’t catch them |  | | 3 | | | | | |  | Mattress on the ground……. | | | | | | | | |  | 3 | | | | | | |
|  |  | |  | | | | | |  | Cot……………………….…… | | | | | | | | |  | 4 | Move on to question 61 | | | | | |
|  |  | |  | | | | | |  | Hammock……………………. | | | | | | | | |  | 5 |  |  |  |  |  |  |
|  |  | |  | | | | | |  | Bedroll……………………….. | | | | | | | | |  | 6 |  |  |  |  |  |  |
|  |  | |  | | | | | |  | On the ground………………. | | | | | | | | |  | 7 |  |  |  |  |  |  |
|  |  | |  | | | | | |  | A bed made of rushes……… | | | | | | | | |  | 8 |  |  |  |  |  |  |
|  |  | |  | | | | | |  | Cardboard……………………. | | | | | | | | |  | 9 |  |  |  |  |  |  |
|  |  | |  | | | | | |  | Rags……………………..……. | | | | | | | | |  | 10 |  |  |  |  |  |  |
|  |  | |  | | | | | |  | Others | |  | | | | | | | | | | | | |  | |
|  | | | | | | | | |  |  | | | | | | | | |  |  | | | | | | |
| **60.** **Do you tend to keep paper, cardboard or rags underneath the mattresses?** | | | | | | | | |  |  | | | | | | | | |  |  | | | | | | |
| Yes……..….…… |  | | 1 | | | | | |  |  | | | | | | | | |  |  | | | | | | |
| No………..…….. |  | | 2 | | | | | |  |  | | | | | | | | |  |  | | | | | | |
|  | | | | | | | | |  |  | | | | | | | | | | | | | | | | |
| **61**. **What does your community do to prevent bugs?** | | | | | | | | |  | **62. What do you do when bugs bite you?** | | | | | | | | | | | | | | | | |
| Apply for help from the local authority ………………..……… | | | | | | |  |  |  | Wash the spot where bitten or apply alcohol……………………………… | | | | | | | | | | | | | |  | | 1 |
|  |  |  |  |  |  |  |  | 1 |  | Take medicine………………….……. | | | | | | | | | | | | | |  | | 2 |
| Fumigation………………….…… | | | | | | |  | 2 |  | Attend the health center…………….. | | | | | | | | | | | | | |  | | 3 |
| Cleaning……………………….… | | | | | | |  | 3 |  | Take animals out of the home……... | | | | | | | | | | | | | |  | | 4 |
| Cutting down the royal palms near the dwellings… | | | | | | |  |  |  | Fumigate……………………………… | | | | | | | | | | | | | |  | | 5 |
|  |  |  |  |  |  |  |  | 4 |  | Clean and tidy the home……….…… | | | | | | | | | | | | | |  | | 6 |
| Nothing………………. | | | | | | |  | 5 |  | Clean the livestock pens……………. | | | | | | | | | | | | | |  | | 7 |
| Don’t know ……………….. | | | | | | |  | 6 |  | Clean the farmyard………………..… | | | | | | | | | | | | | |  | | 8 |
| Others (specify).. | | |  | | | | | |  | I have never been bitten……………. | | | | | | | | | | | | | |  | | 9 |
|  | | |  | | | | | |  | Nothing……………………………..…. | | | | | | | | | | | | | |  | | 10 |
|  | | | | | | |  |  |  | Others (specify) | | | |  | | | | | | | | | | | | |
|  | | | | | | |  |  |  |  | | | |  | | | | | | | | | | | | |
| **63**. **With respect to the royal palms near your dwelling, do you** | | | | | | | | |  |  | | | | | | | | | | | |  |  | | | |
| Cut them?.......…………..……..… | | | | | | |  | 1 |  |  | | | | | | | | | | | |  |  | | | |
| Cut off the leaves or clean them… | | | | | | |  | 2 |  |  | | | | | | | | | | | |  |  | | | |
| I don’t do anything ……………….. | | | | | | |  | 3 |  |  | | | | | | | | | | | |  |  | | | |

***8***. Attitudes

| 64. **When bitten by a bug, who do you attend?** | | | | | | | | | | | | | | | | | | | | | |  | **65. Why?** | | | | | | | | | | | | | | | | | | | | | | | |
| --- | --- | --- | --- | --- | --- | --- | --- | --- | --- | --- | --- | --- | --- | --- | --- | --- | --- | --- | --- | --- | --- | --- | --- | --- | --- | --- | --- | --- | --- | --- | --- | --- | --- | --- | --- | --- | --- | --- | --- | --- | --- | --- | --- | --- | --- | --- |
| Medical…………..….. | | | | | | |  | | | 1 | | | | | Move on to question 66 | | | | | | |  |  |  | | | | | | | | | | | | | | | | | | | |  | | |
| Healer or sorcerer….. | | | | | | |  | | | 2 | | | | | | | | | | | |  |  |  | | | | | | | | | | | | | | | | | | | |  | | |
| Botanist…………….. | | | | | | |  | | | 3 | | | | | | | | | | | |  |  |  | | | | | | | | | | | | | | | | | | | |  | | |
| I don’t attend anyone | | | | | | |  | | | 4 | | | | | | | | | | | |  |  |  | | | | | | | | | | | | | | | | | | | |  | | |
| Others (specify) | | | |  | | | | | | | | | | | | | | | | | |  |  |  | | | | | | | | | | | | | | | | | | | |  | | |
|  | | | | | | | | | | | | | | | | | | | | | |  |  |  | | | | | | | | | | | | | | | | | | | |  | | |
|  | | | | | | | | | | | | | |  | | | |  | | | |  |  | |  | | | | | | | | | | | | | | | | | |  | | | |
| **66**. **Do you participate in the search for bugs in your dwelling and its surroundings?** | | | | | | | | | | | | | | | | | | | | | |  | **67. Why don’t you participate?** | | | | | | | | | | | | | | | | | | | | | | | |
| Always……….. | | | | | | | |  | | | 1 | | | | | Move on to question 68 | | | | | |  |  |  | | | | | | | | | | | | | | | | | | | |  | | |
| Sometimes………… | | | | | | | |  | | | 2 | | | | |  |  |  |  |  |  |  |  |  | | | | | | | | | | | | | | | | | | | |  | | |
| Never………….. | | | | | | | |  | | | 3 | | | | | | | | | | |  |  | | | | | | | | | |  | | |  | | | | | | | | | | |
|  | | | | | | | | | | | | | |  | | | |  | | | |  |  | | | | | | | | | |  | | |  | | | | | | | | | | |
|  | | | | | | | | | | | | | |  | | | |  | | | |  |  | | | | | | | | | |  | | |  | | | | | | | | | | |
| **68.** **Do you agree with spraying insecticide in your home?** | | | | | | | | | | | | | | | | | | | | | |  | **69. Why don’t you agree?** | | | | | | | | | | | | | | | | | | | | | | | |
| Yes… |  | 1 Move on to question 71 | | | | | | | | | | | | | | | | | | |  |  |  |  | | | | | | | | | | | | | | | | | |  | | | | |
| No… |  | 2 | | | | | | | | | | | | | | | | | | | |  |  |  | | | | | | | | | | | | | | | | | |  | | | | |
|  | | | | |  | |  | | | | | | | | | | | | | | |  |  | | | | | | | | | | | | | | | | | | | | | | | |
| **70**. **How long ago was insecticide sprayed in your home?** | | | | | | | | | | | | | | | | | | | | | |  | **71.** **Have you ever talked to any authority of the community about the bugs or Chagas disease?** | | | | | | | | | | | | | | | | | | | | | | | |
| One month ago….. | | | | | |  | | 1 | | | | | | | | | | | | | |  | Yes…….… | | | |  | 1 | | | | | | | | | | | | | | | | | |  |
| Less than one year ago ………………... | | | | | |  | | 2 | | | | | | | | | | | | | |  | No………... | | | |  | 2 | | | | | | | | | | | | | | | | | |  |
| More than one year ago………………… | | | | | |  | | 3 | | | | | | | | | | | | | |  |  | | | | | | | | | | | | | | | | |  | |  | | |  |  |
| Never………. | | | | | |  | | 4 | | | | | | | | | | | | | |  |  | | | | | | | | | | | | | | | | |  | |  | | |  |  |
|  | | | | | | | | | | | | | | | | | | | | | |  |  | | | | | | | | | |  | | |  | | | | | | | | |  |  |
| **72.** **Would you be willing to participate in the surveillance and control measures of the bugs that transmit Chagas disease in your community**? | | | | | | | | | | | | | | | | | | | | | |  | **73**. **What kind of measures would you carry out?** | | | | | | | | | | | | | | | | | | | | | |  |  |
| Yes………………. | | | | | | | | |  | | | 1 | | | | | | | | | |  |  | | |  | | | | | | | | | | | | | | | | |  | |  |  |
| No……………… | | | | | | | | |  | | | 2 | | | | | | | | | |  |  | | |  | | | | | | | | | | | | | | | | |  | |  |  |
|  | | | | | | | | |  | | |  | | | | | | | | | |  |  | | |  | | | | | | | | | | | | | | | | |  | |  |  |
|  | | | | | | | | | | | |  | | | | |  | | | | |  |  | | | | | | | | | | | |  | | |  | | | | | | |  |  |
| **74**. **Who is responsible for the fight against Chagas disease?** | | | | | | | | | | | | | | | | | | | | | |  | **75**. **How do you feel about the prevention and control measures against Chagas disease taken by the Ministry of Health?** | | | | | | | | | | | | | | | | | | | | | |  |  |
| The Ministry of Health | | | | | | | | | | | |  | | | | | 1 | | | | |  | Very satisfied………….. | | | | | | | | | | |  | | | 1 | | | | | | | |  |  |
| Local authorities… | | | | | | | | | | | |  | | | | | 2 | | | | |  | Little satisfied………….. | | | | | | | | | | |  | | | 2 | | | | | | | |  |  |
| The community… | | | | | | | | | | | |  | | | | | 3 | | | | |  | Not satisfied…………... | | | | | | | | | | |  | | | 3 | | | | | | | |  |  |
| Family…………. | | | | | | | | | | | |  | | | | | 4 | | | | |  | I’m not aware of these. | | | | | | | | | | |  | | | 4 | | | | | | | |  |  |
| Everyone................... | | | | | | | | | | | |  | | | | | 5 | | | | |  |  | | | | | | | | | | | |  | | |  | | | | | | |  |  |
| Don’t know ……………… | | | | | | | | | | | |  | | | | | 6 | | | | |  |  | | | | | | | | | | | |  | | |  | | | | | | |  |  |
|  | | | | | | | | | | | | | | | | | | | | | |  |  | | | | | | | | | | | | | | | | | | | | | |  |  |
| **76. If you find bugs in your home, what do you do?** | | | | | | | | | | | | | | | | | | | | | |  | **77.** **Do you use gloves or some kind of protection to catch the bugs?** | | | | | | | | | | | | | | | | | | | | | |  |  |
| Kill them………………… | | | | | | | | | | | | | | | | | | |  | 1 | |  | Yes……….…… | | | | | | |  | | 1 | | | | | | | | | | | | |  |  |
| Burn them…………………. | | | | | | | | | | | | | | | | | | |  | 2 | |  | No…………….. | | | | | | |  | | 2 | | | | | | | | | | | | |  |  |
| Fumigate…………………… | | | | | | | | | | | | | | | | | | |  | 3 | |  |  | | | | | | | | | | | | | | | |  | |  | | | |  |  |
| Take them to the dispensary | | | | | | | | | | | | | | | | | | |  | 4 | |  |  | | | | | | | | | | | | | | | |  | |  | | | |  |  |
| I don’t do anything……………… | | | | | | | | | | | | | | | | | | |  | 5 | |  |  | | | | | | | | | | | | | | | |  | |  | | | |  |  |
|  | | | | | | | | | | | | |  | | | |  | | | | |  |  | | | | | | | | | | | | | | | |  | |  | | | |  |  |
| **78. Would you accept to participate in an educational program concerning Chagas disease?** | | | | | | | | | | | | | | | | | | | | | |  |  | | | | | | | | | | | | | | | | | | | | | |  |  |
| Yes………..… | | |  | 1 | | | | | | | | | | | | | | | | | |  |  | | | | | |  | |  | | | | | | | | | | | | | |  |  |
| No………….. | | |  | 2 | | | | | | | | | | | | | | | | | |  |  | | | | | |  | |  | | | | | | | | | | | | | |  |  |

| Observations: |
| --- |
|  |
|  |
|  |
|  |
|  |
|  |
|  |

Gracias

| Name of the interviewer: |  | | |
| --- | --- | --- | --- |
| Signature of the interviewee: |  | | |
| Date of the interview: |  | | |
|  | Day | Month | Year |
| Duration of the interview: |  |  |  |

Thank you.
