# Supplementary material for: Mixed infections by different Trypanosoma cruzi discrete typing units among Chagas disease patients in an endemic community in Panama
Source: PLoS One. 2020 Nov 12;15(11):e0241921. doi: 10.1371/journal.pone.0241921 (PMC7660484; doi:10.1371/journal.pone.0241921)
Supplement: S1 File — (DOCX) [file pone.0241921.s005.docx]

ASENTIMIENTO INFORMADO

UNIVERSIDAD DE PANAMÁ

**FACULTAD DE MEDICINA**

**DEPATAMENTO DE MICROBIOLOGÍA HUMANA**

**TÍTULO DEL PROYECTO**: “Microbiota y parasitosis intestinal en pacientes panameños infectados con *Trypanosoma cruzi.*

Código de paciente: ____________

Iniciales: __________ Paciente; ______ Testigo

**TITULO DEL PROYECTO:** “Microbiota y parasitosis intestinal en pacientes panameños infectados con *Trypanosoma cruzi*

El Departamento de Microbiología Humana de la Universidad de Panama, el Hospital Santo Tomás y la Universidad de Granada quieren estudiar el parásito que causa la enfermedad de chagas aquí en Panamá. Para ello estamos realizando este estudio.

Si estás de acuerdo en participar, te tomaremos una pequeña muestra de excremento y una de sangre, para ver si tú tienes el parásito que produce esta enfermedad. Puedes sentir algo de temor o un ligero dolor en el momento que se toma la punción venosa . También puedes sangrar un poquito donde se te tome la muestra. Tu participación en este estudio ayudará a comprender mejor esta enfermedad y así poder controlarla.

**¿**Tienes alguna pregunta sobre este estudio de Microbiota y Parasitosis intestinal en enfermos infectados por *Trypanosoma cruzi”?*

Si estás de acuerdo en participar en este estudio, puedes informarnos verbalmente o puedes escribir tu nombre en el espacio abajo.

*__________________________________________________________________*

*Nombre/firma del niño Edad Fecha*

*_______________________________________________________________*

*Firma del testigo Fecha*

*__________________________________________________________________*

##### Firma del testigo (consentimiento verbal) Fecha
