# Supplementary material for: Mixed infections by different Trypanosoma cruzi discrete typing units among Chagas disease patients in an endemic community in Panama
Source: PLoS One. 2020 Nov 12;15(11):e0241921. doi: 10.1371/journal.pone.0241921 (PMC7660484; doi:10.1371/journal.pone.0241921)
Supplement: S2 File — (DOCX) [file pone.0241921.s006.docx]

Informed Consent

University of Panama

Faculty of Medicine

Department of Human Microbiology

**Name of the project: “Microbiota and intestinal parasitosis in Panamese patients infected with Trypanosoma cruzi”**

Code of patient: _______________

Initials: _______________ Patient: _______________ Witness: ______________

The University of Panama together with Santo Tomás Hospital is organizing a study on the microbiota and intestinal parasitosis in patients infected by *Trypanosoma* *cruzi*. Chagas disease is caused by *Trypanosoma* *crursi*, a protozoan parasite, mainly transmitted through the contact with infected excreta of hematophagous triatomine bugs, which, in endemic areas, infect humans and animals.

We would like to invite you to participate in this research project. This is a study that tries to establish new clinical and diagnostic correlations for Panamese chagasic patients. We believe that you could have or suffer Chagas disease, a disease caused by a very small parasite, which is able to cause serious cardiac alterations. There are some tests available to find the parasite and study how your defense against this parasite works. In this study, we are hoping to find new alternatives to carry out a better diagnosis, and, at the same time, we are trying to learn more on how the disease evolves, when other intestinal parasites exist, and its association with the symptomatology. Therefore, it will be necessary to take some fecal as well as blood samples.

If you accept to participate in this study, we will ask you some questions about your health status. We will also carry out a medical examination, and later we would like to take a fecal as well as a blood sample. Therefore, a container will be provided in order to take a small fecal sample and a blood sample will be taken to find out if you have intestinal parasites in your blood. This will be done in the same way as when you consult a doctor for the same reason.

Additionally, a clinical assessment will be made and a series of medical tests will be carried out such as: X-ray of the thorax, a stress test, an electrocardiogram, Doppler and Holter at the cardiology service of Santo Tomás Hospital. All of these procedures and assessments will be carried out under the supervision of a doctor who will be available to answer any question. These procedures are harmless.

The blood sample consists of approximately 10 ml, and will be taken through venipuncture in either arm.

By participating in this study, you will be able to know if you have Chagas disease, so that you will receive adequate care as well as treatment at your health center. There is no cost whatsoever for the participation in this study. Your name will not be used in any report or presentation of the results. The information concerning any case related to the disease will be shared by the medical authorities so that you will receive adequate treatment. The clinical samples obtained will not be used for any purpose not related to this project.

You are free to decide if you wish to decide to participate in the study or not. If you decide to participate and later wish to withdraw, you may do so at any given time.

If you decide to form no longer part of the study or withdraw, you will receive the same attention. You will be able to consult the personnel in charge of the study about any query you may have about this research project. The researchers will inform you about any matter they think might affect you.

Do you have any questions related to the study? If you have any doubt about the study or require any further information, you may contact Msc Alexa C Prescilla, telephone 523-4920, or Dr Azael Saldaña, telephone 523-4920.

**If you have read this consent form or someone has explained it to you, and you agree to participate in the study, please sign below.**

Name of patient: ____________________________

Signature of patient: _________________________

Date: _____________________________________

Name of witness: ____________________________

Signature of witness: ________________________

Date: _____________________________________

Name of researcher__________________________

Signature of researcher: ______________________

Date: _____________________________________
