# Supplementary material for: Mixed infections by different Trypanosoma cruzi discrete typing units among Chagas disease patients in an endemic community in Panama
Source: PLoS One. 2020 Nov 12;15(11):e0241921. doi: 10.1371/journal.pone.0241921 (PMC7660484; doi:10.1371/journal.pone.0241921)
Supplement: S3 File — (DOCX) [file pone.0241921.s007.docx]

CONSENTIMIENTO INFORMADO

UNIVERSIDAD DE PANAMÁ

**FACULTAD DE MEDICINA**

**DEPATAMENTO DE MICROBIOLOGÍA HUMANA**

**TÍTULO DEL PROYECTO**: “Microbiota y parasitosis intestinal en pacientes panameños infectados con *Trypanosoma cruzi*

Código de paciente: ____________

Iniciales: __________ Paciente; ______ Testigo

La Universidad de Panamá en conjunto con el Hospital Santo Tomás y la Universidad de Granada, están organizando un estudio sobre la microbiota y parasitosis intestinal en enfermos infectados por *Trypanosoma cruzi*. La enfermedad de chagas es causada por *Trypanosoma cruzi*, un protozoo parásito transmitido principalmente a través del contacto con las excretas infectadas de chinches triatominos hematófagos, que en las zonas endémicas infecta a los seres humanos y los animales.

Nos gustaría invitarlo a participar en este proyecto de investigación. Este es un estudio que busca hacer nuevas correlaciones clínicas y diagnósticas para los pacientes chagásicos panameños. Pensamos que usted puede tener o padecer la enfermedad de Chagas , una enfermedad que es causada por un parásito muy pequeño que puede causar alteraciones cardiacas importantes. Existen algunas pruebas que se realizan para encontrar el parásito y estudiar cómo usted se defiende contra este parásito. En este estudio esperamos encontrar nuevas alternativas para efectuar un mejor diagnóstico y a la vez estamos tratando de aprender más sobre cómo se comporta la enfermedad, cuando existen otros parásitos intestinales y su asociación con la sintomatología. Para eso es necesario tomar unas muestras de heces y sangre.

Si usted acepta participar en este estudio, le haremos algunas preguntas sobre su estado de salud, también le haremos un examen médico y luego nos gustaría tomar una muestra de heces y de sangre. Para ello se le facilitará un envase para que recoja una pequeña muestra de excremento y se tomará una muestra de sangre, para ver si usted tiene parásitos intestinales y en sangre. Esto se haría de la misma manera que si usted va a otro doctor por la misma razón.

Adicionalmente se le realizara una evaluación clínica y se le realizara una serie de exámenes médicos como: radiografía de tórax, prueba de esfuerzo, electrocardiograma, Doppler y Holter en el servicio de cardiología del Hospital Santo Tomás y en el servicio de radiología del Hospital Regional de Chepo de la CSS.

Todos los procedimientos y evaluaciones serán realizados bajo la supervisión de un médico que estará dispuesto a responder cualquier pregunta. Estos procedimientos son inofensivos.

La muestra de sangre es de aproximadamente 10 ml y será tomada a través de una punción venosa en uno de los brazos.

Con su participación en el estudio usted sabrá si tiene la enfermedad de Chagas para que pueda recibir los cuidados y el tratamiento adecuado en su centro de salud. No existe costo alguno por su participación en este estudio. Su nombre no será usado en ningún reporte o presentación de resultados. La información de algún caso relacionado con la enfermedad será compartida por las autoridades médicas para que usted reciba el tratamiento adecuado. Las muestras clínicas obtenidas no serán utilizadas para ningún otro fin fuera de los alcances de este proyecto.

Usted tiene la libertad de decidir si quiere participar o no en el estudio. Si usted decide participar y luego desea retirarse, lo puede hacer en cualquier momento. Si decide no estar más en el estudio o retirarse del mismo usted recibirá la misma atención. Usted podría consultar con el personal encargado del estudio cualquier pregunta que tuviese acerca de este proyecto de investigación. Los investigadores le informarán a usted cualquier cosa que ellos piensan que le podría afectar.

¿Tiene alguna pregunta respecto al estudio? Si Ud. tiene alguna duda sobre el estudio o si necesita alguna información adicional, puede comunicarse con la Licenciada Alexa C Prescilla al teléfono 523- 4920 o el Dr. Azael Saldaña al teléfono 523-4920.

Si ha leído esta forma de consentimiento o alguien se la explicó y está de acuerdo con su participación en el estudio, por favor firme aquí abajo.

Nombre del paciente:____________________________

Firma del paciente: _____________________________

Fecha: _________________

Nombre del testigo:____________________________

Firma del testigo: _____________________________

Fecha:__________________

Nombre del investigador:____________________________

Firma del investigador: ___________________________

Fecha: _________________
