# Supplementary material for: Mixed infections by different Trypanosoma cruzi discrete typing units among Chagas disease patients in an endemic community in Panama
Source: PLoS One. 2020 Nov 12;15(11):e0241921. doi: 10.1371/journal.pone.0241921 (PMC7660484; doi:10.1371/journal.pone.0241921)
Supplement: S4 File — (DOC) [file pone.0241921.s008.doc]

**UNIVERSIDAD DE PANAMÁ**

**FACULTAD DE MEDICINA**

**DEPATAMENTO DE MICROBIOLOGÍA HUMANA**

**Encuesta de Conocimientos, Actitudes y Prácticas sobre la Enfermedad de Chagas**

**Código de la encuesta:______________________**

**Número de cuestionario:____________________**

**Nombre de la Comunidad:___________________**

El departamento de Microbiología Humana de la Facultad de Medicina de la Universidad de Panamá, está realizando una encuesta para determinar los conocimientos, actitudes y prácticas sobre la enfermedad de Chagas. La información contenida en este formulario que permitiría identificar a cualquier individuo se recolectará con la garantía de que será mantenida en la más estricta confidencialidad y será usada sólo para los propósitos establecidos para este estudio.

***1***. Datos Generales

| 1. Sexo | | | | | | Femenino…… | | | | | | | | |  | | 1 |  | 2. ¿Qué edad tiene en años cumplidos?.. | | | | | | | | | |  |  | |
| --- | --- | --- | --- | --- | --- | --- | --- | --- | --- | --- | --- | --- | --- | --- | --- | --- | --- | --- | --- | --- | --- | --- | --- | --- | --- | --- | --- | --- | --- | --- | --- |
|  | | | | | | Masculino…… | | | | | | | | |  | | 2 |  |  | | | | |  | | | |  | | | |
|  | | |  | | | |  | | | | | | | | | | |  |  | | | | |  | | | |  | | | |
| 3. ¿Qué grado o año escolar más alto aprobó? | | | | | | | | | | | | | | | | | |  | 4. ¿Puede leer y escribir? | | | | | | | | | | | | |
| Ningún grado………… | | | | | | | |  | | 1 | | | | | | | |  | Sí…………. | | | | |  | 1 | | | | | | |
| Primaria………………… | | | | | | | |  | | 2 | | | |  | | | |  | No………… | | | | |  | 2 | | | | | | |
| Secundaria……………… | | | | | | | |  | | 3 | | | | Pase a la pregunta 5 | | | |  |  | | | | |  | | | |  | | | |
| Vocacional..................... | | | | | | | |  | | 4 | | | |  |  |  |  |  |  | | | | |  | | | |  | | | |
| Superior no Universitaria.. | | | | | | | |  | | 5 | | | |  |  |  |  |  |  | | | | |  | | | |  | | | |
| Superior Universitaria…… | | | | | | | |  | | 66 | | | |  |  |  |  |  |  | | | | |  | | | |  | | | |
|  | | | | | | | |  | |  | | | | | | | |  |  | | | | |  | | | |  | | | |
| 5. ¿En el último mes, cuál fue su categoría ocupacional? | | | | | | | | | | | | | | | | | |  | 5a. ¿Cuál es su ingreso semanal actual? | | | | | | | | | | | | |
| Empleado o Asalariado…. | | | | | | | | | | | |  | 1 | | | | |  | Menos de B/. 50.00 | | | | | | |  | | 1 | | | |
| Trabajo por cuenta propia… | | | | | | | | | | | |  | 2 | | | | |  | Entre B/. 51.00 y B/. 100.00 | | | | | | |  | | 2 | | | |
| Ayudante familiar sin remuneración……………… | | | | | | | | | | | |  |  | | | | |  | Entre B/. 101.00 y B/. 150.00 | | | | | | |  | | 3 | | | |
|  |  |  |  |  |  |  |  |  |  |  |  |  | 3 | | | Pase a la pregunta 6 | |  | Más de B/. 151.00 | | | | | | |  | | 4 | | | |
| Trabajo doméstico……….. | | | | | | | | | | | |  | 4 | | |  |  |  |  | | | | | | |  | |  | | | |
| Estudiante………………… | | | | | | | | | | | |  | 5 | | |  |  |  | 5b. ¿Qué ocupación, oficio o trabajo realiza? | | | | | | | | | | | | |
| Desempleado……………… | | | | | | | | | | | |  | 6 | | |  |  |  |  | |  | | | | | | |  | | | |
|  | | | | | | | | |  | |  | | | | | | |  |  | |  | | | | | | |  | | | |
|  | | | | | | | | | | | | | | | | | |  |  | | | | | | | | | | | | |
| 6. ¿Cuántos tiempo tiene de vivir en esta comunidad? | | | | | | | | | | | | | | | | | |  | 7. ¿Cuántas personas viven en su casa? | | | | | | | | | | | | |
|  |  | | | |  | | | | | | | | | | | | |  |  | | |  |  | | | | | | | | |
|  |  | | | |  | | | | | | | | | | | | |  |  | | |  |  | | | | | | | | |
| 8. ¿Cuántos niños (menores de 14 años) viven en la casa? | | | | | | | | | | | | | | | | | |  | 8.a Indíquenos el nombre y la edad del niño | | | | | | | | | | | | |
|  | |  | |  | | | | | | | | | | | | | |  |  | Nombre | | | | | | | Edad | | | | |
|  | | | | | | | | | | | | | | | | | |  |  |  | | | | | | |  | | | | 1 |
|  | | | | | | | | | | | | | | | | | |  |  |  | | | | | | |  | | | | 2 |
|  | | | | | | | | | | | | | | | | | |  |  |  | | | | | | |  | | | | 3 |
|  | | | | | | | | | | | | | | | | | |  |  |  | | | | | | |  | | | | 4 |
|  | | | | | | | | | | | | | | | | | |  |  |  | | | | | | |  | | | | 5 |
|  | | | | | | | | | | | | | | | | | |  |  |  | | | | | | |  | | | | 6 |
|  | | | | | | | | | | | | | | | | | |  |  |  | | | | | | |  | | | | 7 |
|  | | | | | | | | | | | | | | | | | |  |  |  | | | | | | |  | | | | 8 |

***2***. Características de la Vivienda

| 9. ¿De qué material es la mayor parte del techo de la casa? | | | | | | | | | | | |  | | 10. ¿De qué material es la mayor parte de las paredes de la casa? | | | | | | | | | | | | | | | |
| --- | --- | --- | --- | --- | --- | --- | --- | --- | --- | --- | --- | --- | --- | --- | --- | --- | --- | --- | --- | --- | --- | --- | --- | --- | --- | --- | --- | --- | --- |
| Penca o paja…………….. | | | | | |  | 1 | | | | |  | | Bloque, ladrillo, piedra… | | | | | | | | |  | 1 | | | | | |
| Metal (zinc, aluminio)…… | | | | | |  | 2 | | | | |  | | Madera (tablas, troza)…. | | | | | | | | |  | 2 | | | | | |
| Madera…………………… | | | | | |  | 3 | | | | |  | | Quincha………………… | | | | | | | | |  | 3 | | | | | |
| Otros (especifique): | | |  | | | | | | | | |  | | Adobe…………………… | | | | | | | | |  | 4 | | | | | |
|  |  | | | |  | | | | | | |  | | Cañazas………………… | | | | | | | | |  | 5 | | | | | |
|  |  | | | |  | | | | | | |  | | Otros (especifique) | | | | | | | |  | | | | | | | |
|  |  | | | |  | | | | | | |  | |  | |  | | | | | | |  | | | | | | |
|  |  | | | |  | | | | | | |  | |  | |  | | | | | | |  | | | | | | |
| 11. ¿De qué material es la mayor parte del piso de la casa? | | | | | | | | | | | |  | | 12. ¿Cuántos cuartos-dormitorio tiene la casa?............................................. | | | | | | | | | | | | | |  | |
|  | |
| Madera…………… | | | |  | 1 | | | | | | |  | |  | |  | | | | | | |  | | | | | | |
| Tierra……………… | | | |  | 2 | | | | | | |  | | 13. ¿Tiene anexos en su casa? | | | | | | | | | | | | | | | |
| Cemento…………… | | | |  | 3 | | | | | | |  | | Sí……… | |  | | 1 | | | | | | | | | | | |
| Otros (caña, palo)… | | | |  | | | | | | | |  | | No……… | |  | | 2 | | | Pase a pregunta 15 | | | | |  | | | |
|  | | | |  |  | | | | | | |  | |  | |  | |  | | | | | | | | | | | |
|  | | | |  |  | | | | | | |  | |  | |  | |  | | | | | | | | | | | |
| 14. ¿Díganos cuál de estos anexos tiene su vivienda y dónde están ubicados? | | | | | | | | | | | | | | | | | | | | | | | | | | | | | |
| Cocina………………………… | | | | | | | |  | | 1 | | | Alejado de la casa | | | |  | | 1 | Contiguo (menos de 10 m) | | | | | | |  | | 2 |
| Depósito……………………… | | | | | | | |  | | 2 | | | Alejado de la casa | | | |  | | 1 | Contiguo | | | | | | |  | | 2 |
| Gallinero……………………… | | | | | | | |  | | 3 | | | Alejado de la casa | | | |  | | 1 | Contiguo | | | | | | |  | | 2 |
| Porquerizas………………….. | | | | | | | |  | | 4 | | | Alejado de la casa | | | |  | | 1 | Contiguo | | | | | | |  | | 2 |
| Corrales………………………. | | | | | | | |  | | 5 | | | Alejado de la casa | | | |  | | 1 | Contiguo | | | | | | |  | | 2 |
| Baños…………………………. | | | | | | | |  | | 6 | | | Alejado de la casa | | | |  | | 1 | Contiguo | | | | | | |  | | 2 |
| Servicios…………………….. | | | | | | | |  | | 7 | | | Alejado de la casa | | | |  | | 1 | Contiguo | | | | | | |  | | 2 |
| Amontonamiento de leña….. | | | | | | | |  | | 8 | | | Alejado de la casa | | | |  | | 1 | Contiguo | | | | | | |  | | 2 |
| Amontonamiento de piedras.. | | | | | | | |  | | 9 | | | Alejado de la casa | | | |  | | 1 | Contiguo | | | | | | |  | | 2 |
| Otros (especificar): | | |  | | | | | | | | | | Alejado de la casa | | | |  | | 1 | Contiguo | | | | | | |  | | 2 |
|  | | |  | | | | | | | | | |  | | | |  | |  | | |  | | |  | | |  | |
|  | | |  | | | | | | | | | |  | | | |  | |  | | |  | | |  | | |  | |
| 15. ¿Qué fuente de iluminación utiliza en su vivienda? | | | | | | | | | | | | |  | |  | | | | | | | | | | | | | | |
| Velas………………………….. | | | | | | | | |  | | 1 | |  | |  | | | | | | |  | | |  | | |  | |
| Lámparas de kerosén……….. | | | | | | | | |  | | 2 | |  | |  | | | | | | |  | | |  | | |  | |
| Lámparas de batería………… | | | | | | | | |  | | 3 | |  | |  | | | | | | |  | | |  | | |  | |
| Luz eléctrica…………………... | | | | | | | | |  | | 4 | |  | |  | | | | | | |  | | |  | | |  | |
| Otros (especificar): | |  | | | | | | | | | | |  | |  | | | | | | |  | | |  | | |  | |

***3***. Presencia de Animales

| 16. ¿Cuál de los siguientes animales domésticos son de su propiedad? | | | | | | | | | | | | | | | | | | | | | | | | | | | | | | | | |
| --- | --- | --- | --- | --- | --- | --- | --- | --- | --- | --- | --- | --- | --- | --- | --- | --- | --- | --- | --- | --- | --- | --- | --- | --- | --- | --- | --- | --- | --- | --- | --- | --- |
| Gallinas……… | | |  | | | 1 | | | | | | |  | ¿Duermen dentro de la casa? | | | | | Sí | | | |  | | 1 | | | No | | 2 | | 2 |
| Perros……….… | | |  | | | 2 | | | | | | |  | ¿Duermen dentro de la casa? | | | | | Sí | | | |  | | 1 | | | No | |  | | 2 |
| Gatos………… | | |  | | | 3 | | | | | | |  | ¿Duermen dentro de la casa? | | | | | Sí | | | |  | | 1 | | | No | |  | | 2 |
| Cerdos………… | | |  | | | 4 | | | | | | |  | ¿Duermen dentro de la casa? | | | | | Sí | | | |  | | 1 | | | No | |  | | 2 |
| Caballos……… | | |  | | | 5 | | | | | | |  | ¿Duermen dentro de la casa? | | | | | Sí | | | |  | | 1 | | | No | |  | | 2 |
| Patos | | |  | | | 6 | | | | | | |  | ¿Duermen dentro de la casa? | | | | | Sí | | | |  | | 1 | | | No | |  | | 2 |
| Conejos……… | | |  | | | 7 | | | | | | |  | ¿Duermen dentro de la casa? | | | | | Sí | | | |  | | 1 | | | No | |  | | 2 |
| Otros (cuáles): |  | | | | | | | | | | | |  | ¿Duermen dentro de la casa? | | | | | Sí | | | |  | | 1 | | | No | |  | | 2 |
| No tiene | | |  | | | 8 Pase a la pregunta 19 | | | | | | | | | |  | | | | | | | | | | | | | | | | |
|  | | | | | | | | | | | | | | | | | | | | | | | | | | | | | | | | |
| 17. ¿Tiene algunos de estos animales en corrales o jaulas? | | | | | | | | | | | | | | | | |  | 18. ¿A qué distancia de la casa están los animales enjaulados o encorralados? | | | | | | | | | | | | | | |
| Sí………… | |  | | 1 | | | | | | | | | | | | |  | Alejados…… | |  | | | 1 | | | | | | | | | |
| No………… | |  | | 2 | | | | (Pase a la pregunta 19) | | | | | | |  | |  | Contiguo…… | |  | | | 2 | | | | | | | | | |
|  | | | | | | | | | | | | | | | | | | | | | | | | | | | | | | | | |
| 19. ¿Qué animales silvestres ve con frecuencia cerca de su vivienda | | | | | | | | | | | | | | | | |  | 20. ¿Qué animales usted ha visto en las palmas reales? | | | | | | | | | | | | | | |
|  | | | | | | | 1. Si | | | 2. No | |  | | | | |  |  | | | | | | 1. Si | | | 2. No | | | |  | |
| Zarigüeyas o zorras… | | | | | | | | |  |  | 1 | | | | | |  | Zarigüeyas o zorras… | | | | | | | |  |  | | 1 | | | |
| Ardillas……………… | | | | | | | | |  |  | 2 | | | | | |  | Ardillas…………… | | | | | | | |  |  | | 2 | | | |
| Ratones……………… | | | | | | | | |  |  | 3 | | | | | |  | Ratones…………… | | | | | | | |  |  | | 3 | | | |
| Monos……………… | | | | | | | | |  |  | 4 | | | | | |  | Monos…………….. | | | | | | | |  |  | | 4 | | | |
| Aves………………… | | | | | | | | |  |  | 5 | | | | | |  | Aves……………… | | | | | | | |  |  | | 5 | | | |
| Monos Perezosos… | | | | | | | | |  |  | 6 | | | | | |  | Mono perezosos…… | | | | | | | |  |  | | 6 | | | |
| Puerco espín……… | | | | | | | | |  |  | 7 | | | | | |  | Murciélago………….. | | | | | | | |  |  | | 7 | | | |
| Murciélago………… | | | | | | | | |  |  | 8 | | | | | |  | Otros (especifique): | | | |  | | | | | | | | | | |
| Otros (especifique): | | | | |  | | | | | | | | | | | |  |  | | |  | | | | | | | | | | | |

***4***. Vegetación

| 21. ¿Qué tipo de vegetación está cerca (menos de 50 metros) de su casa? | | | | | |  | 22. ¿Qué uso le da usted a las palmas reales o sus derivados? | | | | |
| --- | --- | --- | --- | --- | --- | --- | --- | --- | --- | --- | --- |
| No hay vegetación… | |  | | 1 | |  | Ninguna…………………….. | | |  | 1 |
| Palmas reales……… | |  | | 2 | (Pase a la pregunta 24) |  | Vinos……………………….. | | |  | 2 |
| Árboles frutales…… | |  | | 3 |  |  | Sombra…………………….. | | |  | 3 |
| Arbustos…………… | |  | | 4 |  |  | Pencas……………………… | | |  | 4 |
| Malezas…………… | |  | | 5 |  |  | Leña……………………….. | | |  | 5 |
| Hortalizas………… | |  | | 6 |  |  | Otras (especifique) |  | | | |
| Otras palmas……. | |  | | 7 |  |  |  | | | | |
| Palmas de coco….. | |  | | 8 |  |  |  | | | | |
| Otras (especifique) |  | | | | |  | 23. ¿A qué distancia están las palmas reales de su vivienda? | | | | |
|  | | |  | | |  | Menos de 10 metros…….. | |  | | 1 |
|  | | |  | | |  | Más de 10 metros……….. | |  | | 2 |

***5***. Conocimiento de la Enfermedad de Chagas

| 24. ¿Ha escuchado o conoce qué es la enfermedad de Chagas? | | | | | | | | | | | | | | | | | |  | | 25. ¿A través de qué o quién conoce sobre la enfermedad de Chagas? | | | | | | | | | | | | | | | | | | | | | | | |
| --- | --- | --- | --- | --- | --- | --- | --- | --- | --- | --- | --- | --- | --- | --- | --- | --- | --- | --- | --- | --- | --- | --- | --- | --- | --- | --- | --- | --- | --- | --- | --- | --- | --- | --- | --- | --- | --- | --- | --- | --- | --- | --- | --- |
| Sí…………… |  | | 1 | | | | | | | | | | | | | | |  | | Escuela……………… | | | | | | | | | | |  | | 1 | | | | | | | | | | |
| No…………. |  | | 2 (Pase a la pregunta 34) | | | | | | | | | | | | | | |  | | Radio………………… | | | | | | | | | | |  | | 2 | | | | | | | | | | |
|  |  | |  | | | | | | | | | | | | | | |  | | Periódico………………. | | | | | | | | | | |  | | 3 | | | | | | | | | | |
|  |  | |  | | | | | | | | | | | | | | |  | | Iglesia o culto………… | | | | | | | | | | |  | | 4 | | | | | | | | | | |
|  |  | |  | | | | | | | | | | | | | | |  | | Personal de Salud……… | | | | | | | | | | |  | | 5 | | | | | | | | | | |
|  |  | |  | | | | | | | | | | | | | | |  | | Comités de salud……… | | | | | | | | | | |  | | 6 | | | | | | | | | | |
|  |  | |  | | | | | | | | | | | | | | |  | | Otros (especifique): | | | | | | | |  | | | | | | | | | | | | | | | |
|  |  | |  | | | | | | | | | | | | | | |  | |  | | | | | | | | | | | | |  | |  | | | | | | | | |
| 26. ¿Qué medidas se utilizan en contra de la enfermedad de Chagas? | | | | | | | | | | | | | | | | | |  | | 27. ¿Cómo cree usted que podemos enfermarnos de Chagas? | | | | | | | | | | | | | | | | | | | | | | | |
|  | | | | | | | | | | | | | 1. Sí | | | 2. No | |  | |  | | | | | | | | | | 1. Sí | | | | | | | 2. No | | | | |  | |
| Mantener ordenada la casa….. | | | | | | | | | | | | | |  | |  | 1 | |  | Picadura de un chinche…. | | | | | | | | | | | | | |  | | |  | | | 1 | | | |
| Rociado con insecticida………. | | | | | | | | | | | | | |  | |  | 2 | |  | De persona a persona…… | | | | | | | | | | | | | |  | | |  | | | 2 | | | |
| Evitar animales dentro de la vivienda…………………… | | | | | | | | | | | | | |  | |  |  | |  | Alimentos contaminados… | | | | | | | | | | | | | |  | | |  | | | 3 | | | |
|  |  |  |  |  |  |  |  |  |  |  |  |  |  |  | |  | 3 | |  |  |  |  |  |  |  |  |  |  |  |  |  |  |  |  |  |  |  |  |  |  |  |  |  |
| Mantener ventanas y puertas cerradas en la noche……….. | | | | | | | | | | | | | |  | |  |  | |  | Contaminación con excremento de chinche… | | | | | | | | | | | | | |  | | |  | | | 4 | | | |
|  |  |  |  |  |  |  |  |  |  |  |  |  |  |  | |  | 4 | |  | No sé……………………… | | | | | | | | | | | | | |  | | |  | | | 5 | | | |
| Evitar las palmas reales cerca de las casas………………………… | | | | | | | | | | | | | |  | |  |  | |  | Otras (especifique) | | | | | |  | | | | | | | | | | | | | | | | | |
|  |  |  |  |  |  |  |  |  |  |  |  |  |  |  | |  | 5 | |  |  | | | | | |  | | | | | | | | | | | | | | | | | |
| Evitar el desorden en los alrededores de la casa… ……. | | | | | | | | | | | | | |  | |  |  | |  |  | | | | | |  | | | | | | | | | | | | | | | | | |
|  |  |  |  |  |  |  |  |  |  |  |  |  |  |  | |  | 6 | |  |  | | | | | |  | | | | | | | | | | | | | | | | | |
| Revisar la presencia del chinche periódicamente en la vivienda.…. | | | | | | | | | | | | | |  | |  |  | |  |  | | | | | |  | | | | | | | | | | | | | | | | | |
|  |  |  |  |  |  |  |  |  |  |  |  |  |  |  | |  | 7 | |  |  |  |  |  |  |  |  |  |  |  |  |  |  |  |  |  |  |  |  |  |  |  |  |  |
|  | | |  | | |  | | | | | | | | | | | |  | |  | | |  | | | | | | | | |  | | | | | | | | | | | |
| 28. ¿Le han hecho alguna vez exámenes para el diagnóstico de la enfermedad de Chagas? | | | | | | | | | | | | | | | | | |  | | 29. ¿Tiene usted la enfermedad de Chagas? | | | | | | | | | | | | | | | | | | | | | | | |
| Sí………… | | |  | | 1 | | | | | | | | | | | | |  | | Sí……… | |  | | 1 | | | | | | | | | | | | | | | | | | | |
| No………… | | |  | | 2 | | | | | | | | | | | | |  | | No…….. | |  | | 2 | | | | | | | | | | | | | | | | | | | |
| No sé…….. | | |  | | 3 | | | | | | | | | | | | |  | | No sé…. | |  | | 3 | | | | | | | | | | | | | | | | | | | |
|  | | |  | | |  | | | | | | | | | | | |  | |  | | | | | |  | | |  | | | | | | | | | | | | | | |
| 30. ¿Conoce los síntomas que les da a las personas con la enfermedad de Chagas? | | | | | | | | | | | | | | | | | |  | | 31. ¿Cuáles son estos síntomas? | | | | | | | | | | | | | | | | | | | | | | | |
| Sí…………… | |  | | 1 | | | | | | | | | | | | | |  | | Fiebre…………………… | | | | | | | | | | | | |  | | | | | 1 | | | | | |
| No…………. | |  | | 2 (Pase a la pregunta 32) | | | | | | | | | | | | | |  | | Dolor de cabeza………… | | | | | | | | | | | | |  | | | | | 2 | | | | | |
|  | | | | | | | | | | |  | |  | | | | |  | | Inflamación en los parpados………………… | | | | | | | | | | | | |  | | | | | 3 | | | | | |
|  |  |  |  |  |  |  |  |  |  |  |  |  |  |  |  |  |  |  |  |  |  |  |  |  |  |  |  |  |  |  |  |  |  |  |  |  |  |  |  |  |  |  |  |
|  | | | | | | | | | | |  | |  | | | | |  | | Enfermedad del corazón… | | | | | | | | | | | | |  | | | | | 4 | | | | | |
|  | | | | | | | | | | |  | |  | | | | |  | | Malestar general………… | | | | | | | | | | | | |  | | | | | 5 | | | | | |
|  | | | | | | | | | | |  | |  | | | | |  | | Inflamación en el sitio de la picada…………………….. | | | | | | | | | | | | |  | | | | |  | | | | | |
|  |  |  |  |  |  |  |  |  |  |  |  |  |  |  |  |  |  |  |  |  |  |  |  |  |  |  |  |  |  |  |  |  |  | | | | | 6 | | | | | |
|  | | | | | | | | | | |  | |  | | | | |  | | Otros (especifique) | | | | | |  | | | | | | | | | | | | | | | | | |
|  | | | | | | | | | | |  | |  | | | | |  | |  | | |  | | | | | | | | |  | | | | | | | | | | | |
| 32. ¿Sabe usted que esta enfermedad necesita tratamiento médico? | | | | | | | | | | | | | | | | | |  | | 33. ¿Está usted en tratamiento médico para la enfermedad de Chagas? | | | | | | | | | | | | | | | | | | | | | | | |
| Sí………………… | | | | | | |  | | 1 | | | | | | | | |  | | Sí…………… | | | | |  | | 1 | | | | | | | | | | | | | | | | |
| No…………………… | | | | | | |  | | 2 | | | (Pase a la pregunta 34) | | | | | |  | | No…………… | | | | |  | | 2 | | | | | | | | | | | | | | | | |
|  | | | | | | | |  | |  | | | | | | | |  | |  | | | | | | | | | | | | | | | |  | | | | |  | | |
|  | |  | |  | | | | | | | | | | | | | |  | |  | | | | | | | | | | | | | | | |  | | | | |  | | |
| 34. ¿Sabe usted que esta enfermedad puede ser muy grave y a veces mortal? | | | | | | | | | | | | | | | | | |  | | 35. ¿Algún miembro de su comunidad: | | | | | | | | | | | | | | | | | | | | | | | |
| Sí…………… | |  | | 1 | | | | | | | | | | | | | |  | | está enfermo de Chagas?...... | | | | | | | | | | | | | | | |  | | | 1 | | | | |
| No…………... | |  | | 2 | | | | | | | | | | | | | |  | | ha muerto de Chagas?........... | | | | | | | | | | | | | | | |  | | | 2 | | | | |
|  | | | | | | | | | | |  | |  | | | | |  | | No hay casos……………….. | | | | | | | | | | | | | | | |  | | | 3 | | | | |
|  | | | | | | | | | | | | | | | | | |  | | |  | | | | | | | | | | | | | | |  | | | | | | |  |
| 36. ¿Alguna persona en la vivienda: | | | | | | | | | | | | | | | | | ¿Cuántos? | | | | Parentesco | | | | | | | | | | | | | | | | | | | | | |  |
| está enfermo de Chagas?....... | | | | | | | | | | | | | |  | 1 | | |  | |  |  | | | | | | | | | | | | | | | | | | | | | |  |
| está en tratamiento? | | | | | | | | | | | | | |  | 2 | | |  | |  |  | | | | | | | | | | | | | | | | | | | | | |  |
| ha muerto de Chagas?........... | | | | | | | | | | | | | |  | 3 | | |  | |  |  | | | | | | | | | | | | | | | | | | | | | |  |
| No sabe.................................. | | | | | | | | | | | | | |  | 4 | | |  | |  |  | | | | | | | | | | | | | | | | | | | | | |  |

***6***. Conocimiento del Vector de la Enfermedad de Chagas

| 37. ¿Sabe usted reconocer las heces de los chinches? | | | | | | | | | | |  | 38. ¿Conoce usted que la enfermedad de Chagas se transmite a través de las heces dejadas por los chinches en el sitio de la picada? | | | | | | | | | | | | | | | |
| --- | --- | --- | --- | --- | --- | --- | --- | --- | --- | --- | --- | --- | --- | --- | --- | --- | --- | --- | --- | --- | --- | --- | --- | --- | --- | --- | --- |
| Sí………… |  | 1 | | | | | | | | |  | Sí………… | | | | |  | | 1 | | | | | | | | |
| No………… |  | 2 | | | | | | | | |  | No………… | | | | |  | | 2 | | | | | | | | |
| 39. ¿A cuál de estos reconoce? | | | | | | | | | | |  | 40a. ¿Ha recibido entrenamiento o información para reconocer los chinches? | | | | | | | | | | | | | | | |
| Rhodnius pallescens | | | | | | | | |  | 1 |  | Sí……… | |  | | 1 | | | | | | | | | | | |
| Ninfas de Rhodnius pallescens | | | | | | | | |  | 2 |  | No…….. | |  | | 2 Pase a la pregunta 44 | | | | | | | | | | | |
| Triatoma dimidiata | | | | | | | | |  | 3 |  |  | |  | |  | | | | | | | | | | | |
| Ninfas de Triatoma dimidiata | | | | | | | | |  | 4 |  | 40b. ¿De quién? | |  | | | | | | | | | | | | | |
| Ninguno | | | | | | | | |  | 5 |  |  |  | | | | | | | | | | | | | | |
|  | | | | | | |  | |  | |  |  | |  | |  | | | | | | | | | | | |
| 41. ¿Dónde ha visto los chinches? | | | | | | | | | | |  | 42. ¿A qué hora los ha visto? | | | | | | | | | | | | | | | |
| En el monte…………….. | | | |  | 1 | | | | | |  | Por la mañana…….. | | | | | | | | | |  | | **1** | | | |
| Dentro de la casa……… | | | |  | 2 | | | | | |  | Por la tarde………… | | | | | | | | | |  | | **2** | | | |
| Alrededor de la casa….. | | | |  | 3 | | | | | |  | Por la noche……….. | | | | | | | | | |  | | **3** | | | |
| En la escuela………….. | | | |  | 4 | | | | | |  | A cualquier hora…… | | | | | | | | | |  | | **4** | | | |
| En algunos nidos……… | | | |  | 5 | | | | | |  |  | | | | | | | | |  | |  | | | | |
| Nunca los ha visto…….. | | | |  | 6 | | Pase a la pregunta 44 | | | |  |  | | | | | | | | |  | |  | | | | |
|  | | | |  |  | | | | | |  |  | | | | | | | | |  | |  | | | | |
| 43. ¿En qué lugares conoce usted que viven estos chinches? | | | | | | | | | | |  | 44. ¿Sabe usted de qué se alimentan estos chinches? | | | | | | | | | | | | | | | |
|  | | | | | | 1. Sí | | 2. No |  | |  | Frutas……… | | | | | |  | |  | | | | | | | |
|  |  |  |  |  |  |  |  |  |  |  |  |  |  |  |  |  |  |  | | 1 | | | | | | | |
| En el monte……………… | | | | | |  | |  | 1 | |  | Plantas……… | | | | | |  | | 2 | | | | | | | |
| En las Palmas reales…… | | | | | |  | |  | 2 | |  | Sangre…… | | | | | |  | | 3 | | | | | | | |
| En la Tierra………………. | | | | | |  | |  | 3 | |  | No sabe…… | | | | | |  | | 4 | | | | | | | |
| En madrigueras o nidos… | | | | | |  | |  | 4 | |  | Otras (especifique) | | | | | | |  | | | | | | | | |
| Entre las leñas………….. | | | | | |  | |  | 5 | |  |  | | | | | | | | | | | |  | | | |
| Dentro de la casa……….. | | | | | |  | |  | 6 | |  |  | | | | | | | | | | | | | | | |
| En la cocina………………. | | | | | |  | |  | 7 | |  |  |  |  |  |  |  |  |  |  |  |  |  |  |  |  |  |
| Grietas de las paredes…. | | | | | |  | |  | 8 | |  |  |  |  |  |  |  |  |  |  |  |  |  |  |  |  |  |
| No sé……………………… | | | | | |  | |  | 9 | |  |  | | |  | |  | | | | | | | | | | |
| Debajo de las piedras….. | | | | | |  | |  | 10 | |  |  | | |  | |  | | | | | | | | | | |
| Otras (especifique) | | |  | | | | | | | |  |  | | |  | |  | | | | | | | | | | |
|  | | | | | |  | |  | | |  |  | | | | | | | | | | |  | | |  | |
| 45. ¿Alguna vez usted ha sido picado por estos chinches? | | | | | | | | | | |  | 46. ¿Ha picado este chinche a alguien de su familia? | | | | | | | | | | | | | | | |
| Sí………… |  | 1 | | | | | | | | |  | Sí………… | | | | | |  | | 1 | | | | | | | |
| No………… |  | 2 | | | | | | | | |  | No………… | | | | | |  | | 2 | | | | | | | |
|  |  |  | | | | | | | | |  | No sé…….. | | | | | |  | | 3 | | | | | | | |
|  |  |  | | | | | | | | |  |  | | | | | | | | | | | | |  | |  |
| 47. ¿Sabe usted que los animales domésticos y las ratas también atraen los chinches a la vivienda? | | | | | | | | | | |  |  | | | | | | | | | | | | | | | |
| Sí………… |  | 1 | | | | | | | | |  |  | | | |  | |  | | | | | | | | | |
| No………… |  | 2 | | | | | | | | |  |  | | | |  | |  | | | | | | | | | |

***7***. Prácticas

| 48. ¿Utiliza mosquitero para dormir? | | | | | | | | |  | 49. ¿Quiénes usan mosquitero en está vivienda? | | | | | | | | | | | | | | | | |
| --- | --- | --- | --- | --- | --- | --- | --- | --- | --- | --- | --- | --- | --- | --- | --- | --- | --- | --- | --- | --- | --- | --- | --- | --- | --- | --- |
| Sí……………….. | |  | | 1 | | | | |  | Los adultos……… | | | | |  | | 1 | | | | | | | | | |
| No……………… | |  | | 2 | | | | |  | Los niños………… | | | | |  | | 2 | | | | | | | | | |
| No uso…………. | |  | | 3 | | | | |  | Ambos……………. | | | | |  | | 3 | | | | | | | | | |
|  | |  | |  | | | | |  | Nadie……………….. | | | | |  | | 4 | | | | | | | | | |
|  | | | | | | | | |  |  | | | | | | | | | | | | | | | | |
| 50. ¿Utiliza repelente, loción o mechitas? | | | | | | | | |  | 51. ¿Utiliza mallas metálicas en ventanas y puertas? | | | | | | | | | | | | | | | | |
| Siempre……….. | |  | | 1 | | | | |  | Sí………………. | | | | |  | | | 1 | | | | | | | | |
| A veces………... | |  | | 2 | | | | |  | No……………… | | | | |  | | | 2 | | | | | | | | |
| No usa………… | |  | | 3 | | | | |  |  | | | | |  | | |  | | | | | | | | |
|  | |  | |  | | | | |  |  | | | | |  | | |  | | | | | | | | |
| 52. ¿Con qué periodicidad limpian y ordenan dentro de la vivienda? | | | | | | | | |  | 53. ¿Con que periodicidad limpia y ordena el área alrededor de su vivienda? | | | | | | | | | | | | | | | | |
| Todos los días…… | | | | |  | 1 | | |  | Todos los días……… | | | | | | | | |  | 1 | | | | | | |
| Un día a la semana | | | | |  | 2 | | |  | Un día a la semana…. | | | | | | | | |  | 2 | | | | | | |
| Una vez al mes……. | | | | |  | 3 | | |  | Una vez al mes…….. | | | | | | | | |  | 3 | | | | | | |
| Nunca………………. | | | | |  | 4 | | |  | Nunca……………….. | | | | | | | | |  | 4 | | | | | | |
| Otra…………………. | | | | |  | | | |  | Otra………………. | | | | | |  | | | | | | | | | | |
|  | | | | |  |  | | |  |  | | | | | | | | |  |  | | | | | | |
| 54. ¿Acostumbra pegar o cubrir las paredes de su casa con objetos (calendarios, afiches)? | | | | | | | | |  | 55. ¿Almacena dentro de la casa | | | | | | | | | | | | | | | | |
| Sí………………. | | | | |  | 1 | | |  | Leña?……………… | | | | | | | | |  | 1 | | | | | | |
| No……………… | | | | |  | 2 | | |  | Productos agrícolas? | | | | | | | | |  | 2 | | | | | | |
|  | | | | |  |  | | |  | No almacena nada.... | | | | | | | | |  | 3 | | | | | | |
|  | | | | |  |  | | |  |  | | | | | | | | |  |  | | | | | | |
| 56. ¿Sella los huecos, grietas o rajaduras en las paredes de su vivienda? | | | | | | | | |  | 57. ¿Tiene acumulado materiales de construcción de la vivienda? | | | | | | | | | | | | | | | | |
| Siempre……………. | | | | |  | 1 | | |  | Sí………. |  | | 1 | | | | | | | | | | | | | |
| Nunca….…………… | | | | |  | 2 | | |  | No……… |  | | 2 | | | | | | | | | | | | | |
|  | | | | |  |  | | |  |  | | | | | | | | |  |  | | | | | | |
| 58. ¿En el supuesto de que encuentre chinches, utiliza guantes o algún tipo de protección en las manos para capturarlos? | | | | | | | | |  | 59. ¿En qué duerme usted? | | | | | | | | | | | | | | | | |
| Sí……………… |  | | 1 | | | | | |  | Cama de madera……… | | | | | | | | |  | 1 | | | | | | |
| No…………….. |  | | 2 | | | | | |  | Cama con su colchón… | | | | | | | | |  | 2 | | | | | | |
| No los agarra… |  | | 3 | | | | | |  | Colchón sobre el piso… | | | | | | | | |  | 3 | | | | | | |
|  |  | |  | | | | | |  | Catre…………………… | | | | | | | | |  | 4 | Pase a la pregunta 61 | | | | | |
|  |  | |  | | | | | |  | Hamaca………………. | | | | | | | | |  | 5 |  |  |  |  |  |  |
|  |  | |  | | | | | |  | Petate………………….. | | | | | | | | |  | 6 |  |  |  |  |  |  |
|  |  | |  | | | | | |  | Suelo…………………… | | | | | | | | |  | 7 |  |  |  |  |  |  |
|  |  | |  | | | | | |  | Cama de carricillas…… | | | | | | | | |  | 8 |  |  |  |  |  |  |
|  |  | |  | | | | | |  | Cartones………………. | | | | | | | | |  | 9 |  |  |  |  |  |  |
|  |  | |  | | | | | |  | Trapos…………………. | | | | | | | | |  | 10 |  |  |  |  |  |  |
|  |  | |  | | | | | |  | Otros | |  | | | | | | | | | | | | |  | |
|  | | | | | | | | |  |  | | | | | | | | |  |  | | | | | | |
| 60. ¿Acostumbra a colocar papeles, cartones o trapos debajo de los colchones? | | | | | | | | |  |  | | | | | | | | |  |  | | | | | | |
| Sí……………… |  | | 1 | | | | | |  |  | | | | | | | | |  |  | | | | | | |
| No…………….. |  | | 2 | | | | | |  |  | | | | | | | | |  |  | | | | | | |
|  | | | | | | | | |  |  | | | | | | | | | | | | | | | | |
| 61. ¿Qué hace la comunidad para evitar los chinches? | | | | | | | | |  | 62. ¿Qué hace usted cuando le pican los chinches? | | | | | | | | | | | | | | | | |
| Solicitar apoyo a las autoridades locales………………………… | | | | | | |  |  |  | Se lava o pone alcohol en la picada… | | | | | | | | | | | | | |  | | 1 |
|  |  |  |  |  |  |  |  | 1 |  | Toma medicamentos…………………. | | | | | | | | | | | | | |  | | 2 |
| Fumigación…………………… | | | | | | |  | 2 |  | Acude al centro de salud…………….. | | | | | | | | | | | | | |  | | 3 |
| Limpieza……………………… | | | | | | |  | 3 |  | Saca los animales de la casa……….. | | | | | | | | | | | | | |  | | 4 |
| Eliminación de palmas reales cercanas a las viviendas…… | | | | | | |  |  |  | Fumiga………………………………… | | | | | | | | | | | | | |  | | 5 |
|  |  |  |  |  |  |  |  | 4 |  | Limpia y ordena la casa……………… | | | | | | | | | | | | | |  | | 6 |
| Nada…………………………. | | | | | | |  | 5 |  | Limpia de corrales……………………. | | | | | | | | | | | | | |  | | 7 |
| No sé………………………….. | | | | | | |  | 6 |  | Limpia el patio………………………… | | | | | | | | | | | | | |  | | 8 |
| Otras (Especifique)... | | |  | | | | | |  | Nunca me han picado……………….. | | | | | | | | | | | | | |  | | 9 |
|  | | |  | | | | | |  | Nada……………………………………. | | | | | | | | | | | | | |  | | 10 |
|  | | | | | | |  |  |  | Otras (especifique)… | | | |  | | | | | | | | | | | | |
|  | | | | | | |  |  |  |  | | | |  | | | | | | | | | | | | |
| 63. ¿Con respecto a las palmas reales cercanas a la vivienda, usted | | | | | | | | |  |  | | | | | | | | | | | |  |  | | | |
| las tumba?...............…………… | | | | | | |  | 1 |  |  | | | | | | | | | | | |  |  | | | |
| desfoliar o limpia……………..…. | | | | | | |  | 2 |  |  | | | | | | | | | | | |  |  | | | |
| No hace nada………………….. | | | | | | |  | 3 |  |  | | | | | | | | | | | |  |  | | | |

***8***. Actitudes

| 64. ¿De ser picado por un chinche acudiría al? | | | | | | | | | | | | | | | | | | | | | |  | 65. ¿Por qué? | | | | | | | | | | | | | | | | | | | | | | | |
| --- | --- | --- | --- | --- | --- | --- | --- | --- | --- | --- | --- | --- | --- | --- | --- | --- | --- | --- | --- | --- | --- | --- | --- | --- | --- | --- | --- | --- | --- | --- | --- | --- | --- | --- | --- | --- | --- | --- | --- | --- | --- | --- | --- | --- | --- | --- |
| Médico……………….. | | | | | | |  | | | 1 | | | | | Pase a pregunta 66 | | | | | | |  |  |  | | | | | | | | | | | | | | | | | | | |  | | |
| Curandero o Brujo….. | | | | | | |  | | | 2 | | | | | | | | | | | |  |  |  | | | | | | | | | | | | | | | | | | | |  | | |
| Botánico…………….. | | | | | | |  | | | 3 | | | | | | | | | | | |  |  |  | | | | | | | | | | | | | | | | | | | |  | | |
| No iría a ningún lado. | | | | | | |  | | | 4 | | | | | | | | | | | |  |  |  | | | | | | | | | | | | | | | | | | | |  | | |
| Otros (especifique) | | | |  | | | | | | | | | | | | | | | | | |  |  |  | | | | | | | | | | | | | | | | | | | |  | | |
|  | | | | | | | | | | | | | | | | | | | | | |  |  |  | | | | | | | | | | | | | | | | | | | |  | | |
|  | | | | | | | | | | | | | |  | | | |  | | | |  |  | |  | | | | | | | | | | | | | | | | | |  | | | |
| 66. ¿Participa en la búsqueda de chinches en su vivienda y los alrededores? | | | | | | | | | | | | | | | | | | | | | |  | 67. ¿Por qué no participa? | | | | | | | | | | | | | | | | | | | | | | | |
| Siempre…………….. | | | | | | | |  | | | 1 | | | | | Pase a la pregunta 68 | | | | | |  |  |  | | | | | | | | | | | | | | | | | | | |  | | |
| A veces……………… | | | | | | | |  | | | 2 | | | | |  |  |  | | | | | | | | | | | | | | | | | | | |  | | |
| Nunca……………….. | | | | | | | |  | | | 3 | | | | | | | | | | |  |  | | | | | | | | | |  | | |  | | | | | | | | | | |
|  | | | | | | | | | | | | | |  | | | |  | | | |  |  | | | | | | | | | |  | | |  | | | | | | | | | | |
|  | | | | | | | | | | | | | |  | | | |  | | | |  |  | | | | | | | | | |  | | |  | | | | | | | | | | |
| 68. ¿Está de acuerdo con el rociado de insecticida en su casa? | | | | | | | | | | | | | | | | | | | | | |  | 69. ¿Por qué no está de acuerdo? | | | | | | | | | | | | | | | | | | | | | | | |
| Sí……… |  | 1 (Pase a la pregunta 71) | | | | | | | | | | | | | | | | | | |  |  |  |  | | | | | | | | | | | | | | | | | |  | | | | |
| No…… |  | 2 | | | | | | | | | | | | | | | | | | | |  |  |  | | | | | | | | | | | | | | | | | |  | | | | |
|  | | | | |  | |  | | | | | | | | | | | | | | |  |  | | | | | | | | | | | | | | | | | | | | | | | |
| 70. ¿Hace cuánto tiempo rociaron con insecticida su casa? | | | | | | | | | | | | | | | | | | | | | |  | 71. ¿Alguna vez ha hablado con alguna autoridad de la comunidad sobre los chinches o la enfermedad de Chagas? | | | | | | | | | | | | | | | | | | | | | | | |
| Un mes………….. | | | | | |  | | 1 | | | | | | | | | | | | | |  | Sí………… | | | |  | 1 | | | | | | | | | | | | | | | | | |  |
| Menos de un año… | | | | | |  | | 2 | | | | | | | | | | | | | |  | No……….. | | | |  | 2 | | | | | | | | | | | | | | | | | |  |
| Más de un año…… | | | | | |  | | 3 | | | | | | | | | | | | | |  |  | | | | | | | | | | | | | | | | |  | |  | | |  | |
| Nunca……………. | | | | | |  | | 4 | | | | | | | | | | | | | |  |  | | | | | | | | | | | | | | | | |  | |  | | |  | |
|  | | | | | | | | | | | | | | | | | | | | | |  |  | | | | | | | | | |  | | |  | | | | | | | | |  | |
| 72. ¿Estaría dispuesto a participar de la vigilancia y control de los chinches que transmiten la enfermedad de Chagas en la comunidad? | | | | | | | | | | | | | | | | | | | | | |  | 73. ¿Qué tipo de acciones llevaría a cabo? | | | | | | | | | | | | | | | | | | | | | |  | |
| Sí………………………. | | | | | | | | |  | | | 1 | | | | | | | | | |  |  | | |  | | | | | | | | | | | | | | | | |  | |  | |
| No……………………… | | | | | | | | |  | | | 2 | | | | | | | | | |  |  | | |  | | | | | | | | | | | | | | | | |  | |  | |
|  | | | | | | | | |  | | |  | | | | | | | | | |  |  | | |  | | | | | | | | | | | | | | | | |  | |  | |
|  | | | | | | | | | | | |  | | | | |  | | | | |  |  | | | | | | | | | | | |  | | |  | | | | | | |  | |
| 74. ¿La responsabilidad de luchar contra la enfermedad de Chagas es de? | | | | | | | | | | | | | | | | | | | | | |  | 75. ¿Cómo se siente con las acciones de prevención y control de la enfermedad de Chagas del Ministerio de Salud? | | | | | | | | | | | | | | | | | | | | | |  | |
| El Ministerio de Salud… | | | | | | | | | | | |  | | | | | 1 | | | | |  | Muy Satisfecho……. | | | | | | | | | | |  | | | 1 | | | | | | | |  | |
| Autoridades locales…… | | | | | | | | | | | |  | | | | | 2 | | | | |  | Poco satisfecho……. | | | | | | | | | | |  | | | 2 | | | | | | | |  | |
| De la comunidad……… | | | | | | | | | | | |  | | | | | 3 | | | | |  | Nada satisfecho……. | | | | | | | | | | |  | | | 3 | | | | | | | |  | |
| De la familia……………. | | | | | | | | | | | |  | | | | | 4 | | | | |  | No las conoce………. | | | | | | | | | | |  | | | 4 | | | | | | | |  | |
| De todos......................... | | | | | | | | | | | |  | | | | | 5 | | | | |  |  | | | | | | | | | | | |  | | |  | | | | | | |  | |
| No sabe………………… | | | | | | | | | | | |  | | | | | 6 | | | | |  |  | | | | | | | | | | | |  | | |  | | | | | | |  | |
|  | | | | | | | | | | | | | | | | | | | | | |  |  | | | | | | | | | | | | | | | | | | | | | |  | |
| 76. ¿Sí encuentras chinches en su casa que hace con ellos? | | | | | | | | | | | | | | | | | | | | | |  | 77. ¿Utiliza guantes o algún tipo de protección en las manos para agarrar los chinches? | | | | | | | | | | | | | | | | | | | | | |  | |
| Los mata……………………… | | | | | | | | | | | | | | | | | | |  | 1 | |  | Sí……………… | | | | | | |  | | 1 | | | | | | | | | | | | |  | |
| Los quema……………………. | | | | | | | | | | | | | | | | | | |  | 2 | |  | No…………….. | | | | | | |  | | 2 | | | | | | | | | | | | |  | |
| Fumiga………………………… | | | | | | | | | | | | | | | | | | |  | 3 | |  |  | | | | | | | | | | | | | | | |  | |  | | | |  | |
| Los lleva al puesto de salud… | | | | | | | | | | | | | | | | | | |  | 4 | |  |  | | | | | | | | | | | | | | | |  | |  | | | |  | |
| No hace nada………………… | | | | | | | | | | | | | | | | | | |  | 5 | |  |  | | | | | | | | | | | | | | | |  | |  | | | |  | |
|  | | | | | | | | | | | | |  | | | |  | | | | |  |  | | | | | | | | | | | | | | | |  | |  | | | |  | |
| 78. ¿Aceptaría participar en un programa educativo sobre la enfermedad de Chagas? | | | | | | | | | | | | | | | | | | | | | |  |  | | | | | | | | | | | | | | | | | | | | | |  | |
| Sí……………… | | |  | 1 | | | | | | | | | | | | | | | | | |  |  | | | | | |  | |  | | | | | | | | | | | | | |  | |
| No…………….. | | |  | 2 | | | | | | | | | | | | | | | | | |  |  | | | | | |  | |  | | | | | | | | | | | | | |  | |

| Observaciones: |
| --- |
|  |
|  |
|  |
|  |
|  |
|  |
|  |

Gracias

| Nombre del entrevistador: |  | | |
| --- | --- | --- | --- |
| Firma de la entrevista: |  | | |
| Fecha de la entrevista: |  | | |
|  | Día | Mes | Año |
| Duración de la entrevista |  |  |  |

GRACIAS
