# Supplementary material for: Mixed infections by different Trypanosoma cruzi discrete typing units among Chagas disease patients in an endemic community in Panama
Source: PLoS One. 2020 Nov 12;15(11):e0241921. doi: 10.1371/journal.pone.0241921 (PMC7660484; doi:10.1371/journal.pone.0241921)
Supplement: S5 File — (DOCX) [file pone.0241921.s009.docx]

Informed consent

University of Panama

Faculty of Medicine

Department of Human Microbiology

Project name: “Microbiota and intestinal parasitosis in Panamese patients infected with *Trypanosoma cruzi”*

Code of patient: _________________

Initials: ____________ Patient: __________ Witness: _____________

The Department of Human Microbiology of University of Panama, Santo Tomás Hospital and University of Granada, would like to study the parasite that causes Chagas disease here in Panama. Therefore, we are carrying out this study.

If you agree to participate, a small fecal sample as well as a blood sample will be taken to find out if you have the parasite that produces the disease. It might happen that you will feel a little pain at the moment when the venipuncture is made. Also, you may bleed a little at the point when the sample is taken. Your participation in this study will help to better understand this disease and, thus, control it.

Do you have any question about this study on the Microbiota and the intestinal parasitosis in patients infected with *Trypanosoma cruzi*

If you agree to participate in this study, you can either inform us verbally or you can write your name in the space provided below.

Name/signature of the child Age Date

*_____________________ _____ ____________*

*Signature of the witness Date*

*_________________________ _______________*

*Signature of the witness (verbal consent) Date*

*_______________________ ______________*
